# Safety and protective efficacy of PfSPZ Vaccine administered to HIV-negative and -positive Tanzanian adults

**DOI:** 10.1172/JCI169060

**Published:** 2024-01-09

**Authors:** Said Jongo, L.W. Preston Church, Florence Milando, Munira Qassim, Tobias Schindler, Mohammed Rashid, Anneth Tumbo, Gloria Nyaulingo, Bakari M. Bakari, Thabit Athuman Mbaga, Latipha Mohamed, Kamaka Kassimu, Beatus S. Simon, Maxmillian Mpina, Irfan Zaidi, Patrick E. Duffy, Phillip A. Swanson, Robert Seder, Jonathan D. Herman, Maanasa Mendu, Yonatan Zur, Galit Alter, Natasha KC, Pouria Riyahi, Yonas Abebe, Tooba Murshedkar, Eric R. James, Peter F. Billingsley, B. Kim Lee Sim, Thomas L. Richie, Claudia Daubenberger, Salim Abdulla, Stephen L. Hoffman

**Affiliations:** 1 Ifakara Health Institute (IHI), Bagamoyo, Tanzania.; 2 Sanaria Inc., Rockville, Maryland, USA.; 3 Swiss Tropical Public Health Institute, Basel, Switzerland.; 4 University of Basel, Basel, Switzerland.; 5 Laboratory of Malaria Immunology and Vaccinology and; 6 Vaccine Research Center, NIH, Bethesda, Maryland, USA.; 7 Division of Infectious Disease, Brigham and Women’s Hospital, Boston, Massachusetts, USA.; 8 The Ragon Institute of MGH, MIT and Harvard, Cambridge, Massachusetts, USA.; 9 Protein Potential LLC, Rockville, Maryland, USA.

**Keywords:** Infectious disease, Vaccines, Adaptive immunity, Malaria

## Abstract

**BACKGROUND:**

Sanaria PfSPZ Vaccine, composed of attenuated *Plasmodium falciparum* (Pf) sporozoites (SPZ), protects against malaria. We conducted this clinical trial to assess the safety and efficacy of PfSPZ Vaccine in HIV-positive (HIV^+^) individuals, since the HIV-infection status of participants in mass vaccination programs may be unknown.

**METHODS:**

This randomized, double-blind, placebo-controlled trial enrolled 18- to 45-year-old HIV-negative (HIV^–^) and well-controlled HIV^+^ Tanzanians (HIV viral load <40 copies/mL, CD4 counts >500 cells/μL). Participants received 5 doses of PfSPZ Vaccine or normal saline (NS) over 28 days, followed by controlled human malaria infection (CHMI) 3 weeks later.

**RESULTS:**

There were no solicited adverse events in the 9 HIV^–^ and 12 HIV^+^ participants. After CHMI, 6 of 6 NS controls, 1 of 5 HIV^–^ vaccinees, and 4 of 4 HIV^+^ vaccinees were Pf positive by quantitative PCR (qPCR). After immunization, anti–Pf circumsporozoite protein (anti-PfCSP) (isotype and IgG subclass) and anti-PfSPZ antibodies, anti-PfSPZ CD4^+^ T cell responses, and Vδ2^+^ γδ CD3^+^ T cells were nonsignificantly higher in HIV^–^ than in HIV^+^ vaccinees. Sera from HIV^–^ vaccinees had significantly higher inhibition of PfSPZ invasion of hepatocytes in vitro and antibody-dependent complement deposition (ADCD) and Fcγ3B binding by anti-PfCSP and ADCD by anti–cell-traversal protein for ookinetes and SPZ (anti-PfCelTOS) antibodies.

**CONCLUSIONS:**

PfSPZ Vaccine was safe and well tolerated in HIV^+^ vaccinees, but not protective. Vaccine efficacy was 80% in HIV^–^ vaccinees (*P* = 0.012), whose sera had significantly higher inhibition of PfSPZ invasion of hepatocytes and enrichment of multifunctional PfCSP antibodies. A more potent PfSPZ vaccine or regimen is needed to protect those living with HIV against Pf infection in Africa.

**TRIAL REGISTRATION:**

ClinicalTrials.gov NCT03420053.

**FUNDING:**

Equatorial Guinea Malaria Vaccine Initiative (EGMVI), made up of the Government of Equatorial Guinea Ministries of Mines and Hydrocarbons, and Health and Social Welfare, Marathon Equatorial Guinea Production Limited, Noble Energy, Atlantic Methanol Production Company, and EG LNG; Swiss government, through ESKAS scholarship grant no. 2016.0056; Intramural Research Program of the National Institute of Allergy and Infectious Diseases, NIH; NIH grant 1U01AI155354-01.

## Abstract 2

PfSPZ Vaccine is safe but not protective against P. falciparum infections in persons living with HIV.

## Graphical abstract

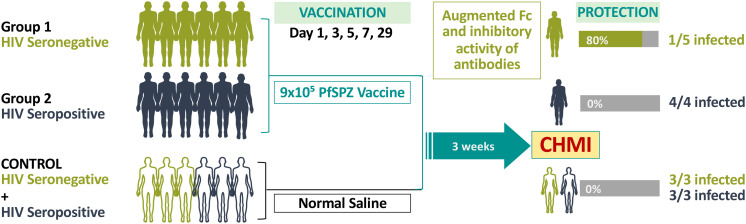

## Introduction

Our long-term goal is the development of a malaria vaccine against *Plasmodium falciparum* (Pf) malaria that can be used in endemic areas for mass vaccination programs (MVPs) to immunize the entire population, halt transmission, and eliminate the parasite (1). To be appropriate for use in MVPs, the vaccine must be well tolerated and safe for all recipients, even those who are immunocompromised, and ideally administered over a short interval with a high level of protective efficacy.

Sanaria PfSPZ Vaccine, composed of radiation-attenuated, aseptic, purified, cryopreserved Pf sporozoites (SPZ), is a candidate for MVPs. It has been assessed in more than 2,000 research subjects 5 months to 61 years old in 22 clinical trials in the US, Europe, 6 countries in Africa, and Indonesia (2–21). PfSPZ Vaccine has been well tolerated and safe at all doses and inoculation intervals tested. A metaanalysis of 13 double-blind, placebo-controlled trials of PfSPZ Vaccine, 11 in Africa, showed no significant difference in adverse event (AE) profiles between vaccinees and controls who received normal saline (NS) (22). Vaccine efficacy (VE) has reached 100% against homologous (same Pf strain as the vaccine, NF54) controlled human malaria infection (CHMI) at 3 to 7 weeks after the last dose of vaccine (3, 14, 19) and 78% to 80% against heterologous (Pf7G8 strain) CHMI at 3 and 9 to 10 weeks (6, 20), with protection lasting for at least 14 months against homologous (5) and 8 months against heterologous (7) CHMI in malaria-naive participants. VE against Pf infection in field studies in African adults, who mount less robust vaccine-induced immune responses, has been demonstrated to last for at least 18 months and range from 47% to 86% (8, 21, 23). Multidose priming of malaria-naive participants, in whom 2 or 4 doses of PfSPZ Vaccine were administered in the first week, provided better VE than other immunization regimens (15, 18, 20) and allowed shortened administration regimens, improving practicality for use in MVPs.

Our primary target is fielding a vaccine for malaria elimination in Africa. An estimated 25.4 million people living with HIV reside in the WHO African region (24), where 98% of Pf infections also occur (25). Demonstrating safety in HIV-infected individuals is critical to the PfSPZ Vaccine–development program, as it would allow immunization of an entire population without screening for HIV-infection status. HIV-infected individuals, however, frequently demonstrate impaired cellular immune responses, even when CD4^+^ T cell counts are indistinguishable from those found in those without HIV infection (26). It is unclear whether PfSPZ Vaccine, even if safe and well tolerated, would offer benefit to persons living with HIV, since cellular immunity is thought to underlie the protective immunity induced by this vaccine (2). Antibody responses, although associated with protection (8, 17, 19) and potentially contributory, have not been considered critical for durable immunity (1, 2). The current clinical trial was designed to assess the safety, tolerability, immunogenicity, and VE of a multidose prime regimen of PfSPZ Vaccine administered over 28 days in HIV-positive (HIV^+^) compared with HIV-negative (HIV^–^) individuals.

## Results

### Study participants

**Figure 1:**
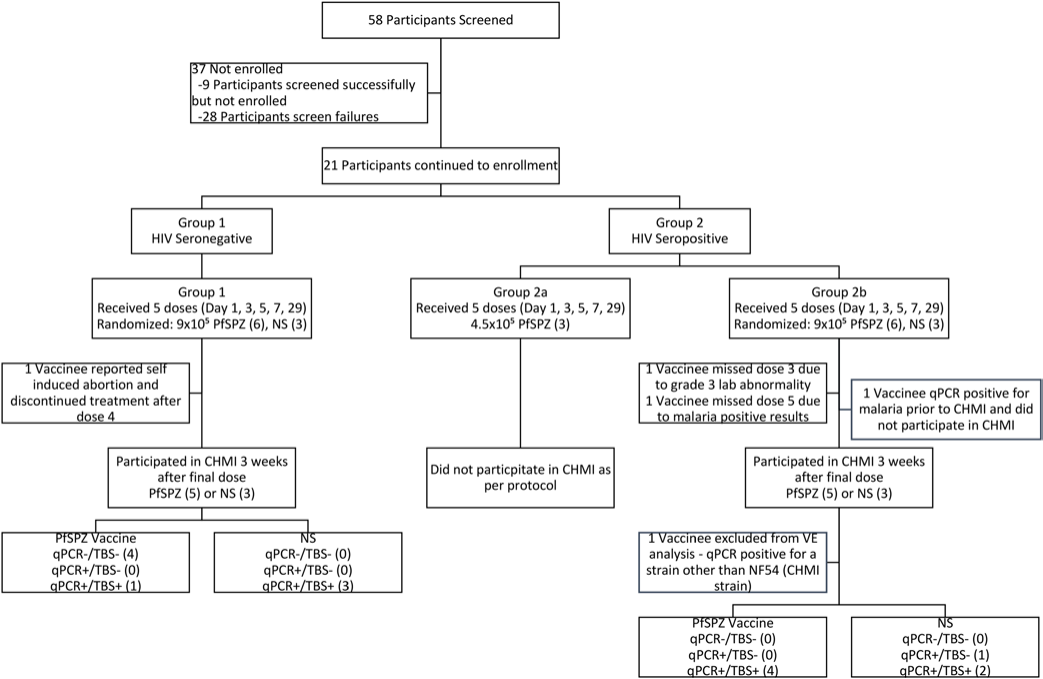
CONSORT flow diagram. Reasons for screen failure can be found in Supplemental Table 3.

Study participants were scheduled to receive a 5-dose regimen of Sanaria PfSPZ Vaccine (9.0 × 10^5^ PfSPZ of PfSPZ Vaccine per dose) or NS administered by direct venous inoculation (DVI) on days 1, 3, 5, 7, and 29. Both products were clear, colorless, odor free, administered as 0.5 mL, and indistinguishable by study staff or participants, enabling a randomized, double-blind design. Nine HIV^–^ participants (6 male, 3 female; median age, 25 years) were enrolled first into group 1 and randomized to receive PfSPZ Vaccine (*n* = 6) or NS (*n* = 3) (Figure 1). After vaccine safety was demonstrated, 12 HIV^+^ participants were enrolled. Three participants were allocated to a reduced-dose, open-label pilot safety cohort (group 2a) receiving 4.5 × 10^5^ PfSPZ of PfSPZ Vaccine, after which 9 participants (6 male, 3 female; median age, 40 years) were randomized to receive 9.0 × 10^5^ PfSPZ of PfSPZ Vaccine (*n* = 6) or NS (*n* = 3) (group 2b). Further details on participant demographics, the medical history for each HIV^+^ participant, and the reasons for screening failure are presented in Supplemental Tables 1–3 (supplemental material available online with this article; https://doi.org/10.1172/JCI169060DS1). HIV^–^ participants receiving the 9.0 × 10^5^ PfSPZ dose were younger (median age, 25.5; range, 18–33 years) than the HIV^+^ participants receiving the same dose (median age, 41.5; range, 30–43 years).

### Parasitemia during vaccination

**Figure 2:**
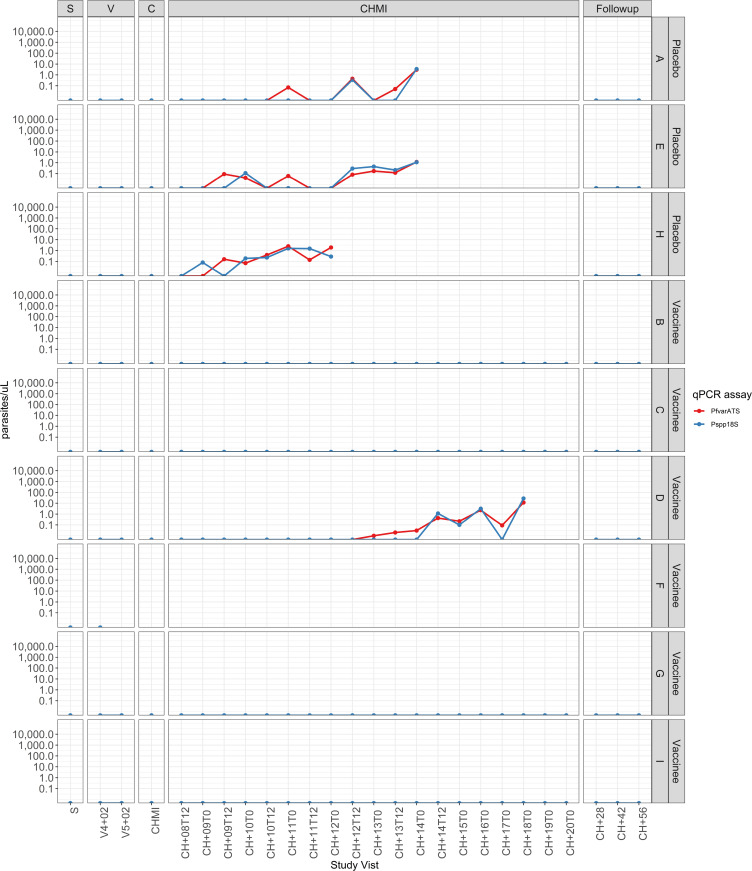
Detection of parasitemia by qPCR at screening, during vaccination and before, during, and after CHMI in HIV^+^ participants. Three HIV^+^ participants (J, K, and L) did not undergo CHMI and are not included in this figure. Participant S withdrew prior to CHMI. Participant N was excluded from analysis after genotyping demonstrated the parasitemia detected during the CHMI period was not NF54, the challenge strain. S, screening; V, vaccination; C, CHMI,

Study participants were recruited from the town of Bagamoyo in northern, coastal Tanzania. The surrounding Bagamoyo district is considered an area of moderate Pf malaria transmission, although risk is uneven throughout the district, with substantial variability observed in malaria risk indicators such as antenatal clinic prevalence rate (2.0%), annual parasite incidence (7.24%), and Pf prevalence in 5- to 16-year-old schoolchildren (73.6%) (27). Participants were screened by thick blood smear (TBS) and quantitative PCR (qPCR) for asexual blood-stage parasitemia prior to immunization, and none were positive. Immunizations were initiated without presumptive malaria treatment of the study participants to clear any latent, undetected parasitemia. Two IV^+^ participants were diagnosed with Pf infection during the immunization period. One was a control participant with asymptomatic parasitemia by qPCR 2 days after the fourth dose of NS, who was treated with artemether/lumefantrine (AL) and confirmed negative by qPCR at the time of the fifth dose of NS and before CHMI (Figure 2, participant M). The second was a vaccinee who presented with asymptomatic parasitemia by TBS (257 Pf/μL) prior to the fifth dose of PfSPZ Vaccine. The participant was treated with AL, was not administered the fifth dose, and underwent CHMI on schedule 3 weeks later (Figure 2, participant N). This sample was not available for microsatellite testing (Supplemental Table 4), although a subsequent qPCR-positive sample obtained during the CHMI period did not match NF54.

### Safety

#### AEs following immunization.

Solicited local AEs at the site of injection were recorded for 2 days and solicited systemic AEs for 7 days after each immunization (Supplemental Table 5). No solicited local or systemic AEs were reported in any of the participants receiving PfSPZ Vaccine or NS. Unsolicited AEs were recorded for 2 weeks after each immunization. Three grade-1 and 2 grade-2 unsolicited AEs were reported in 5 participants receiving PfSPZ Vaccine: 4 HIV^–^ participants experienced abdominal pain, a superficial burn, contact dermatitis, and vaginal bleeding, respectively, the latter developing into a serious AE (SAE) (see below), and 1 HIV^+^ participant experienced grade 2 diarrhea. One grade 1 unsolicited AE (lower abdominal pain) was reported in an HIV^–^ control (Supplemental Table 6). All unsolicited AEs were deemed unrelated to the study product.

One SAE was reported in an HIV^–^ participant who was hospitalized for vaginal bleeding and incomplete abortion after the self-administration of misoprostol. The participant had been withdrawn from further immunization a few days prior for a positive serum pregnancy test prior to the scheduled fifth immunization. The participant recovered without complications, and the SAE was deemed unrelated to study participation. Based upon this history, the participant likely conceived 4 days prior to the informed consent process and screening evaluation (6 days prior to first immunization), at which time serum pregnancy test had been negative.

#### Laboratory abnormalities following immunization.

Hematology and biochemistry (alanine transaminase [ALT], aspartate aminotransferase [AST], total bilirubin, and serum creatinine) were obtained prior to each of the 5 immunizations, 2 and 7 days after the fourth immunization and the fifth immunization. Although grade-1 and -2 leukopenia and neutropenia were observed more frequently in the HIV^+^ vaccinees and controls, there were no significant differences in the number of participants experiencing laboratory abnormalities between HIV^–^ and HIV^+^ vaccinees who received the 9 × 10^5^ PfSPZ dose (Supplemental Table 7). Grade 3 neutropenia (580 cells/μL) was observed in 1 HIV^+^ participant 2 days after the second dose of PfSPZ Vaccine. The third dose was not administered due to this finding, but as the neutrophil count rose, the fourth dose was administered 2 days later. Two days after the fourth dose of PfSPZ Vaccine, the absolute neutrophil count was 1,030 cells/μL (lower limit of normal 1,180 cells/μL) and remained above 1,000 cells/μL for the duration of the trial.

HIV viral load remained below the lower limits of quantitation (40 copies/mL) for all participants at all time points throughout immunizations and CHMI. No changes were noted in the CD4^+^ lymphocyte counts 3 weeks after the priming series of immunizations or 29 days after the booster among the 3 HIV^+^ participants who served as a pilot safety group (Supplemental Figure 1A). Among the 9 HIV^+^ participants from group 2b who received sequential doses of 9.0 × 10^5^ PfSPZ or NS, there was no consistent pattern to suggest CD4^+^ lymphocyte counts or percentages declined in response to immunization (Supplemental Figure 1, B and C).

#### AEs following administration of PfSPZ Challenge (through day +6).

CHMI was performed by DVI of 3.2 × 10^3^ PfSPZ of Sanaria PfSPZ Challenge, a product identical to PfSPZ Vaccine using the same West African–derived parasite strain (NF54), except that the parasites are not irradiated and are therefore fully infectious (4). The PfSPZ Challenge parasites develop in the liver during the first 5 to 6 days after injection, before breaking out into the blood. One HIV^+^ participant reported solicited AEs (arthralgias, fatigue, and headache) attributed to PfSPZ Challenge 6 days after administration (Supplemental Table 8). This participant was consistently qPCR negative until 15 days after CHMI (Figure 2, participant Q).

### VE

PfSPZ Challenge was administered by DVI 3 weeks after the last booster dose of vaccine (except for 1 participant who missed the pre-CHMI booster dose but received CHMI on schedule; see below). Participants were followed daily by TBS starting 7 days (day 8) after injection. If TBS was positive, qPCR was run to confirm the result prior to treatment, and if qPCR was positive, participants were then treated with a 3-day course of AL using directly observed treatment (DOT). VE was defined as 1-relative risk and computed by comparing the number of vaccinees developing parasitemia to the combined control groups.

#### NS controls.

**Figure 3:**
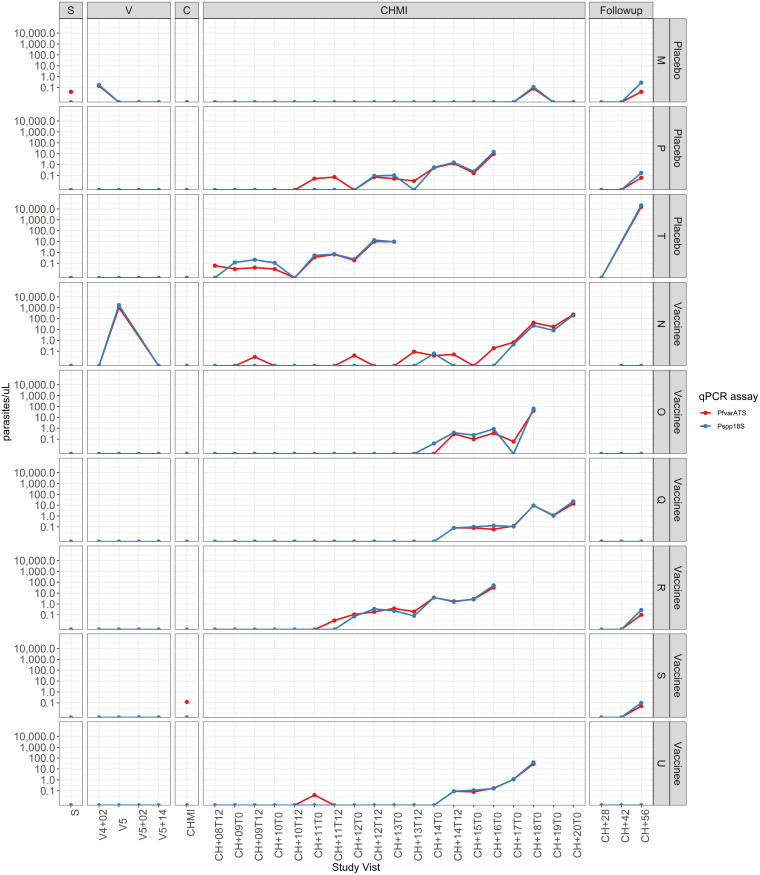
Detection of parasitemia by qPCR at screening, during vaccination, and before, during, and after CHMI in HIV^–^ participants. Results for the PlasQ qPCR target PfvarATS are in red; results for pan-plasmodium 18s rDNA are in blue.

**Figure 4:**
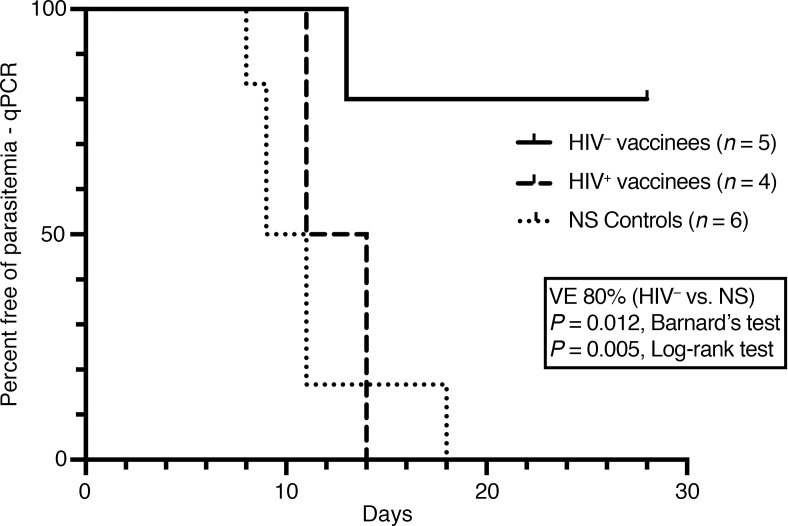
Time to parasitemia in vaccinees and controls participating in CHMI as assessed by qPCR. All 5 HIV^–^ vaccinees received 9.0 × 10^5^ PfSPZ on days 1, 3, 5, 7, and 29. Three HIV^+^ vaccinees received 9.0 × 10^5^ PfSPZ on days 1, 3, 5, 7, and 29; 1 HIV^+^ vaccinee received 9.0 × 10^5^ PfSPZ on days 1, 3, 7, and 29. Three weeks after the last vaccination, participants underwent homologous CHMI.

Three of 3 HIV^–^ and 3 of 3 HIV^+^ controls participated in CHMI (Figure 1). Six of 6 developed parasitemia by qPCR (Figure 2, Figure 3, and Figure 4) at a median of 10 days (9, 9, and 11 days for HIV^–^ and 8, 11, and 18 days for HIV^+^) after CHMI. Five of 6 were positive by TBS on days 12, 12, and 14 for HIV^–^ participants and on days 13 and 16 for the HIV^+^ participants.

#### Group 1: HIV^–^ PfSPZ Vaccine recipients.

Five of 6 PfSPZ Vaccine recipients received 5 immunizations with 9.0 × 10^5^ PfSPZ on days 1, 3, 5, 7, and 29 and participated in CHMI (Figure 1). Four of 5 were negative by both qPCR and TBS (Figure 3 and Figure 4). One of 5 was positive by qPCR (13 days after CHMI) and TBS (18 days after CHMI). VE 3 weeks after the last dose of vaccine was 80% (4 of 5 vaccinees versus 0 of 6 controls negative by qPCR, *P* = 0.012, Barnard’s test, 2-tailed).

#### Group 2b: HIV^+^ PfSPZ Vaccine recipients.

Four of 6 participants received 5 immunizations with 9.0 × 10^5^ PfSPZ on days 1, 3, 5, 7, and 29 (Figure 1). One participant was qPCR positive for Pf just prior to the scheduled CHMI and did not participate in CHMI; the other 3 participants all developed parasitemia by qPCR and TBS (Figure 4). One participant received 4 immunizations with 9.0 × 10^5^ PfSPZ on days 1, 3, 7, and 29 (the third dose was held for asymptomatic grade 3 neutropenia), participated in CHMI, and was subsequently positive by qPCR and TBS (Figure 4). One participant received 4 immunizations with 9.0 × 10^5^ PfSPZ on days 1, 3, 5, and 7, was TBS positive (257 Pf/μL) without symptoms prior to the fifth dose, received a full course of AL, and proceeded to CHMI without the fifth dose. This participant developed parasitemia by qPCR on day 9 (Figure 2, participant N) and was TBS positive on day 20. The Pf genotype of this infection (Supplemental Table 4), however, did not match NF54, and this participant was excluded from the VE analysis. The median prepatent period for the remaining 4 participants by qPCR (Figure 4) was 12.5 (range 11, 14) days after CHMI. VE 3 weeks after the last dose of vaccine was 0% (0 of 4 vaccinees versus 0 of 6 controls negative by qPCR, *P* = 1.0, Barnard’s test, 2-tailed). VE was also significantly different between HIV^–^ and HIV^+^ vaccinees (4 of 5 versus 0 of 4 negative by qPCR, *P* = 0.014, Barnard’s test, 2-tailed).

### Parasitemia and symptoms during and after CHMI (day +7 onwards)

Seven to 28 days after administration of PfSPZ Challenge, clinical manifestations attributable to malaria (fever, chills, headache, fatigue, malaise, myalgia, arthralgia, nausea, or vomiting) were reported in 6 of 12 participants positive by qPCR (6 of 11 positive by TBS), including 1 HIV^–^ participant (NS control) and 5 HIV^+^ participants (3 PfSPZ and 2 NS). The most frequently reported symptom was headache, occurring in all 6 participants (Supplemental Table 8). Grade 3 fever was reported in 1 HIV^–^ control, while all other events were grade 2 or less. All events resolved within 12 hours after the start of antimalarial treatment. None of the qPCR-negative participants reported symptoms.

One day prior to the last scheduled study visit (55 days after CHMI and 40 days after completion of AL administered by DOT), 1 of the HIV^+^ NS control participants presented with fever (axillary temperature 38.0°C) and was TBS positive (23,951 Pf/μL) (Figure 2, participant T). Parasite genotyping strongly suggested that this participant had PfNF54 parasitemia 12.5 days after CHMI (which was subsequently treated) and acquired new, unrelated Pf infection 55 days after CHMI (Supplemental Table 4). The other 2 HIV^+^ NS controls and 1 of the 5 HIV^+^ vaccinees, all of whom had been treated for PfNF54 parasitemia during CHMI, were determined to have Pf parasitemia by qPCR without symptoms on day 56 after CHMI (Figure 2, participants M, P, and R). One additional HIV^+^ vaccinee was qPCR positive at the same study visit (Figure 2, participant S); this participant was qPCR positive just prior to CHMI, was treated with AL, and did not undergo CHMI, but was followed to the end of the study. These 4 participants were retreated with 6 doses of AL administered by DOT, followed for 55 to 60 additional days (to day 111 or 116 after CHMI), then confirmed negative by qPCR. While it is presumed that all 4 infections were naturally acquired, each had very low estimated parasite densities by the more sensitive PlasQ qPCR (range 0.040 to 0.1 copies/μL) and it was not possible to perform parasite genotyping successfully.

### Antibody responses

Antibodies were assessed in sera from participants from all groups prior to the first immunization and 2 weeks after the final immunization (Supplemental Table 9).

#### IgG and IgM antibodies to PfCSP by ELISA.

**Figure 5:**
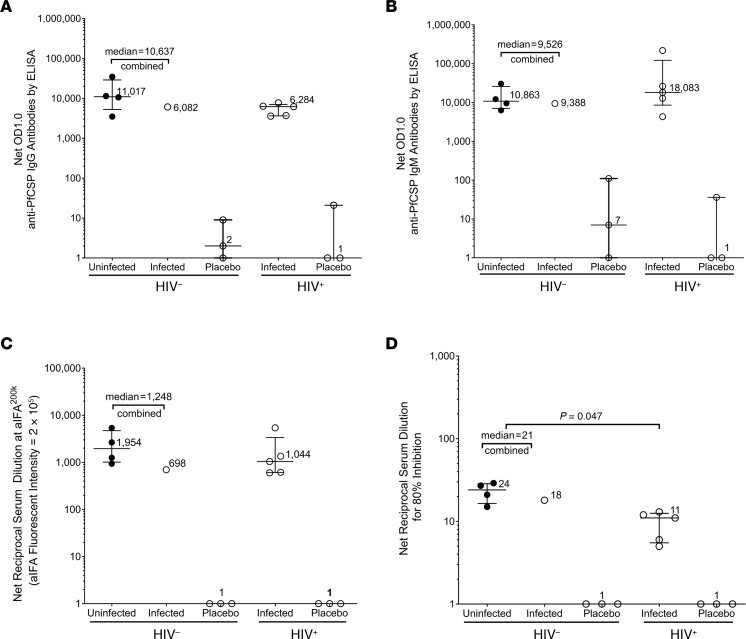
Antibodies to PfCSP and PfSPZ and functional activity of sera against PfSPZ. Antibodies and functional activity assessed in sera taken 2 weeks after the final dose of PfSPZ Vaccine in participants who were uninfected (protected) (black circles) and infected (white circles) during homologous CHMI with PfSPZ Challenge (NF54) administered 3 weeks after the final dose. Median and interquartile range of net OD 1.0 for IgG (**A**) and IgM (**B**) antibodies to PfCSP by ELISA. Median and interquartile range of net IgG antibodies to PfSPZ by aIFA (**C**). Net serum dilution for 80% inhibition of PfSPZ invasion of hepatocytes by aISI (**D**). There were no significant differences between HIV^–^ and HIV^+^ vaccinees for antibodies to PfCSP by ELISA or PfSPZ by aIFA. However, the serum dilution at which there was 80% inhibition of PfSPZ invasion of hepatocytes (aISI) was significantly higher in HIV^–^ vaccinees than in HIV^+^ vaccinees (*P* = 0.047, Wilcoxon-Mann-Whitney test adjusted for multiple comparisons).

All 10 participants (5 HIV^–^ who received all 5 doses and 5 HIV^+^, 2 of whom only received 4 doses) who received PfSPZ Vaccine followed by CHMI met our criteria for development of IgG and IgM antibodies to Pf circumsporozoite protein (PfCSP) (seroconversion) (Supplemental Table 10); none of the 6 NS control participants met these criteria. Net anti-PfCSP IgG and IgM antibodies were substantially higher for vaccinees than placebo controls for HIV^–^ and HIV^+^ participants for each of the assays. IgG antibodies to PfCSP trended lower in the HIV^+^ vaccinees than in the HIV^–^ vaccinees, whereas IgM antibodies trended higher in the HIV^+^ vaccinees compared with the HIV^–^ vaccinees, but the differences were not significant (*P* = 0.86 for both comparisons) (Figure 5, A and B).

#### IgG antibodies to PfSPZ by automated immunofluorescence assay.

All (5 of 5) HIV^–^ (100%) and 4 of 5 HIV^+^ (80%) participants who received PfSPZ Vaccine, but none of the 6 NS control participants, met our criteria for development of antibodies to PfSPZ (Supplemental Table 10). Net anti-PfSPZ IgG antibodies were higher for vaccinees than for the placebo controls for HIV^–^ and HIV^+^ participants. IgG antibodies to PfSPZ trended lower in the HIV^+^ vaccinees than in the HIV^–^ vaccinees, but the differences were not significant (*P* = 0.86) (Figure 5C).

#### Functional activity of sera by automated inhibition of sporozoite invasion assay.

All (5 of 5) HIV^–^ (100%) and 4 of 5 HIV^+^ (80%) participants who received PfSPZ Vaccine, but none of the 6 NS control participants, met our criteria for developing inhibition of sporozoite invasion (ISI) activity (Supplemental Table 10). Net serum dilutions at which there was 80% inhibition were higher for vaccinees than for controls for HIV^–^ and HIV^+^ participants. The net serum dilutions for 80% inhibition in the ISI, adjusted for multiple comparisons, was significantly higher in the HIV^–^ vaccinees (21) than in the HIV^+^ vaccinees (11) (*P* = 0.047) (Figure 5D).

#### IgG antibodies to PfMSP5 by ELISA.

Only 1 participant met our criteria for development of antibodies to Pf merozoite protein 5 (PfMSP5). This HIV^+^ individual received 4 doses of PfSPZ Vaccine, on days 1, 3, 5, and 7. The subject did not receive the fifth dose because naturally acquired Pf parasitemia was detected on day 29 (Supplemental Table 10 and Supplemental Figure 2). Antibodies to PfMSP5 were detected on day 41.

#### Systems serology analysis.

**Figure 6:**
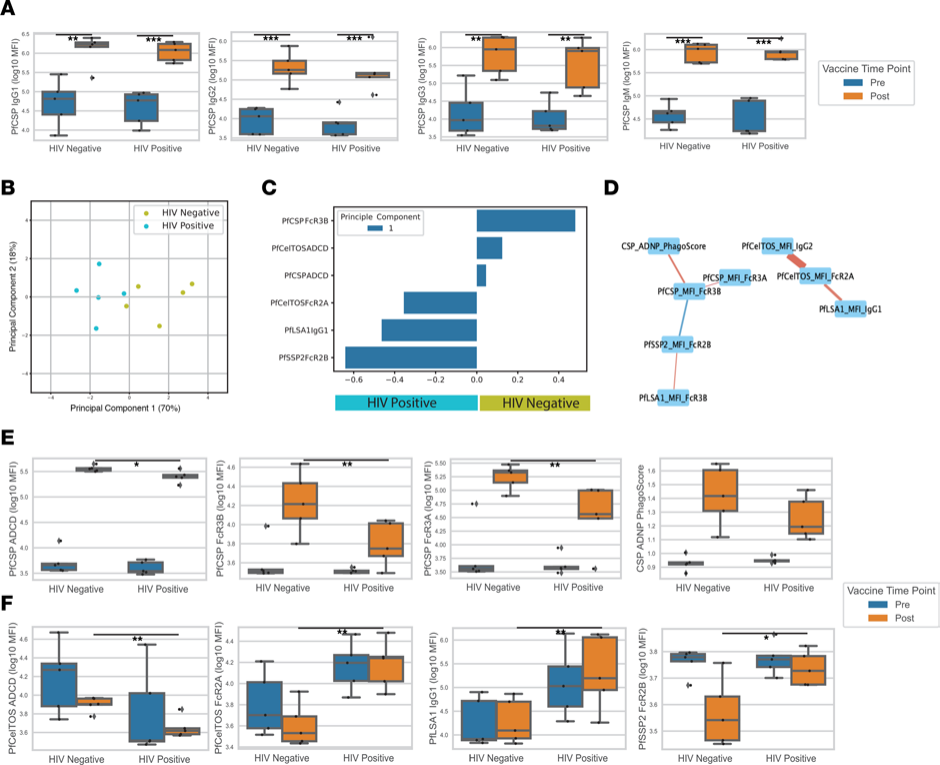
Systems serology analysis. (**A**) Box plots of PfCSP isotype and levels of IgG1, IgG2, IgG3, and IgM in prevaccination (blue) and postvaccination (orange) samples for HIV^–^ and HIV^+^ vaccinees. (**B**) PCA plot of postvaccination HIV^+^ (cyan) and HIV^–^ (green) participants based on the elastic-net selected features (α = 0.75). Principle component 1 explained 70% of the variation observed. (**C**) Bar plot of the loadings for PC1. Features with a positive value were enriched in HIV^–^ individuals, and features with a negative value were enriched in HIV^+^ individuals. (**D**) Elastic-net selected features cocorrelation network. All measured features that had a Spearman’s *r* > 0.5 and *P* < 0.2 with 1 of the selected features was included in this network plot. The color of an edge connotes the direction of correlation (red edge = +; blue edge = –), and the width of an edge connotes the significance of the relationship (wide edge = more significant; thin edge = less significant). (**E**) Box plots of CSP elastic-net selected and cocorrelated features from **C** and **D** of prevaccination (blue) and postvaccination (orange) samples for HIV^–^ and HIV^+^ vaccinees. (**F**) Box plots of elastic-net of non-CSP antibody features of prevaccination (blue) and postvaccination (orange) samples for HIV^–^ and HIV^+^ vaccinees. Mann-Whitney *U* method was used for statistical testing (**A**, **E**, and **F**). *P < 0.1; **P < 0.05; *** *P* < 0.01.

Since we saw a difference in the automated ISI (aISI) between HIV^+^ and HIV^–^ vaccinees, but failed to find significant differences in levels of IgG or IgM antibodies to PfCSP (Figure 5, A and B) or PfSPZ (Figure 5C), we hypothesized that non–titer-driven differences in antibody profiles might be implicated in the lack of protection of HIV^+^ subjects. To determine whether the HIV status of vaccinees altered the anti-Pf humoral immune responses and potentially explained the differences in VE, we measured antibody isotype, IgG subclass, Fc receptor–binding (FcR-binding) profiles, and complement activity using 7 Pf proteins expressed in SPZ and/or liver-stage parasites (Supplemental Figure 4). The antigens were PfCSP, sporozoite surface protein 2/thrombospondin-related anonymous protein (PfSSP2/PfTRAP), apical membrane antigen 1 (PfAMA1), exported protein 1 (PfEXP1), liver-stage antigen 1 (PfLSA1), cell-traversal protein for ookinetes and SPZ (PfCelTOS), and erythrocyte binding antigen 175 (PfEBA-175) (Supplemental Figure 3). HIV^–^ vaccinees did not develop significantly higher IgA, IgM, IgG1, IgG2, IgG3, or IgG4 antibodies to any of the malaria antigens compared with HIV^+^ vaccinees (Figure 6A), supporting the ELISA data showing that differences in the levels of antibodies between HIV^–^ and HIV^+^ vaccinees could not explain the differences in VE.

To further investigate how humoral profiles differed between the 2 groups, we performed a multivariate analysis. Using elastic-net to identify the core antibody features among the high dimensional humoral antibody profiles, our principal component analysis (PCA) separated HIV^–^ and HIV^+^ vaccinees primarily based on principal component 1 (PC1) (Figure 6B). Separation seen in PC1 was primarily driven by PfCSP and PfCelTOS functional antibody enrichment in HIV^–^ vaccinees, specifically antibody-dependent complement deposition (ADCD) activity and Fcγ3B binding of PfCSP antibodies and ADCD activity of PfCelTOS antibodies (Figure 6C). A network consisted of strongly correlated features of PfCSP functional attributes, including PfCSP Fcγ3B, Fcγ3A, and antibody-dependent neutrophil phagocytosis (ADNP), that were enriched in HIV^–^ vaccinees (Figure 6D). PfCSP Fcγ3B, Fcγ3A, and ADCD were each significantly enriched in HIV^–^ as compared with HIV^+^ vaccinees, while PfCSP ADNP trended toward enrichment (Figure 6E), demonstrating that the functional anti-PfCSP antibody response was diminished in HIV^+^ vaccinees. These findings suggest that lower functional anti-PfCSP antibody responses may have contributed to the decrease in VE found in HIV^+^ vaccinees.

On the other hand, PfCelTOS Fcγ2A, PfLSA1 IgG1, and PfSSP2/TRAP Fcγ2B features were enriched in HIV^+^ vaccinees (Figure 6C). Cocorrelation analysis revealed a network of the PfCelTOS, PfLSA1, and PfSSP2/TRAP features that were enriched in HIV^+^ vaccinees without additional close correlations observed (Figure 6D). Though PfCelTOS ADCD, PfCelTOS Fcγ2A, PfLSA1 IgG1, and PfSSP2 Fcγ2B had significant differences between HIV^+^ and HIV^–^ vaccinees, the prevaccination time points were similar or higher than the postvaccination time points (Figure 6F). This suggested that the differences observed for these features, largely enriched in HIV^+^ vaccinees, were less likely to be due to a response to vaccination. Taken together, these findings suggest that PfSPZ vaccination in HIV^–^ individuals resulted in higher functional anti-PfCSP antibody responses that potentially contributed to the greater VE in HIV^–^ vaccinees.

### T cell responses to PfSPZ

**Figure 7:**
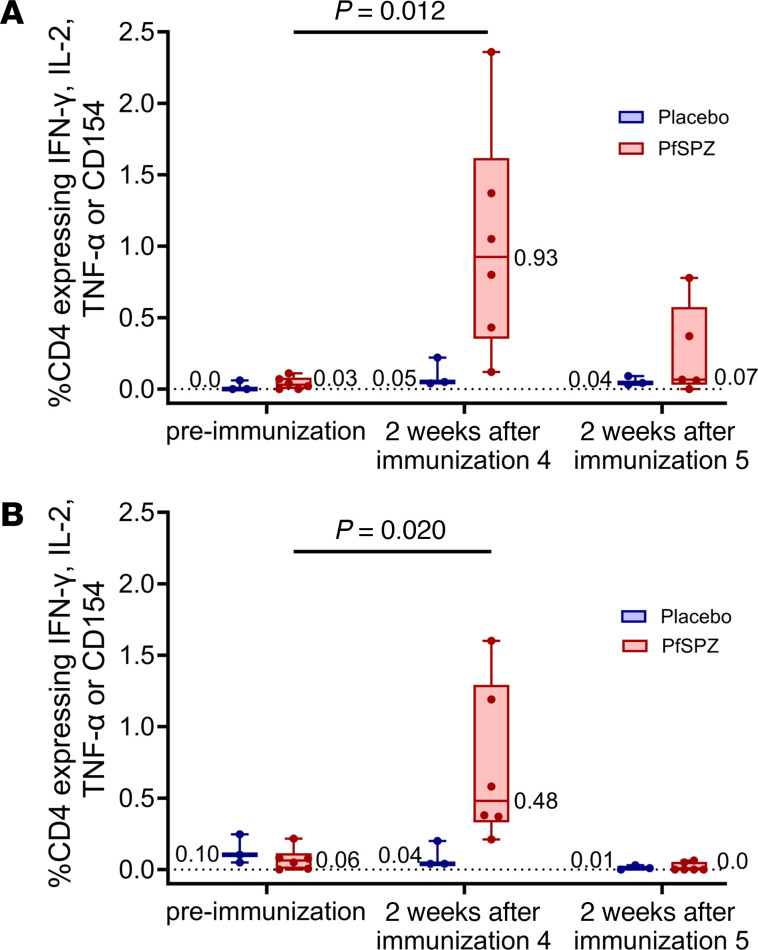
PfSPZ-specific memory CD4^+^ T cell responses before and after immunization. Percentages of memory CD4^+^ T cells in the blood expressing IFN-γ, IL-2, TNF-α, or CD154 at preimmunization or 2 weeks after the fourth and fifth doses of PfSPZ Vaccine (9.0 × 10^5^) in HIV^–^ (**A**) and HIV^+^ (**B**) participants. Results show the percentages of cytokine-producing cells after incubation with PfSPZ minus the percentages of cytokine-producing cells after incubation with vaccine diluent (medium with 1% human serum albumin). Bars indicate median values within each group, and circles indicate individual participant data. The number of CD4^+^ T cells expressing IFN-γ, IL-2, TNF-α, or CD154 2 weeks after the initial series of 4 immunizations was significantly higher than before immunization (*P* = 0.012, HIV^–^; *P* = 0.020, HIV^+^, Kruskal-Wallis test), but not significantly different between HIV^–^ and HIV^+^ participants (*P* = 0.59). Cytokine expression 2 weeks after the final immunization in vaccinees was not significantly different from before immunization (*P* = 0.54, HIV^–^; *P* = 0.70, HIV^+^, Wilcoxon-Mann-Whitney test adjusted for multiple comparisons).

T cell responses to PfSPZ were assessed by polychromatic flow cytometry on cryopreserved PBMCs acquired prior to immunization, 2 weeks after the fourth scheduled dose of PfSPZ Vaccine and 2 weeks after the fifth scheduled dose of vaccine in participants who received 9.0 × 10^5^ PfSPZ of PfSPZ Vaccine per dose. The percentages of memory CD4^+^ T cells producing IFN-γ, IL-2, or TNF-α or expressing CD154 in response to PfSPZ stimulation (Figure 7 and Supplemental Figure 6) 2 weeks after the initial priming immunizations were significantly higher than before immunization (HIV^–^, *P* = 0.012; HIV^+^, *P* = 0.020; Kruskal-Wallis test). Cytokine expression 2 weeks after the final immunization in vaccinees, however, declined and was not significantly different from before immunization (HIV^–^, *P* = 0.54; HIV^+^, *P* = 0.70). There was no significant difference in the increase in median frequency of cytokine-producing memory CD4^+^ T cells between the HIV^–^ vaccinees and the HIV^+^ vaccinees (*P* = 0.423, Kruskal-Wallis test), although the median percentage of expression was higher in HIV^–^ (0.92) than in HIV^+^ (0.48) vaccinees. PfSPZ-specific CD8^+^ T cell responses 2 weeks after the fourth and fifth immunizations were undetectable for all but 2 vaccinees, 1 HIV^–^ and 1 HIV^+^ (Supplemental Figures 5 and 6).

**Figure 8:**
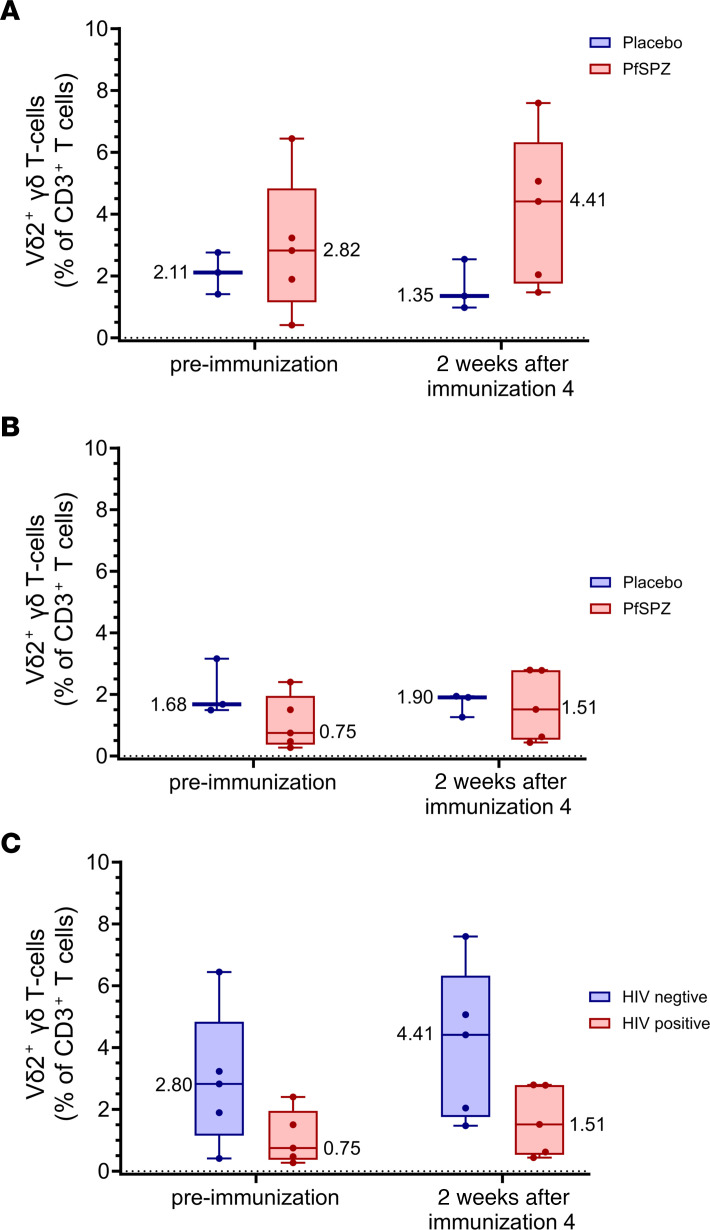
Vδ2^+^ γδ T cells before immunization and after fourth immunization. The percentages of CD3^+^ T cells that were Vδ2^+^ before immunization or 2 weeks after the fourth dose of PfSPZ Vaccine (9.0 × 10^5^) or NS in HIV^–^ (**A**) and HIV^+^ (**B**) participants. Results show the percentages of unstimulated CD3^+^ T cells staining positive for Vδ2. Bars indicate median values within each group, and circles indicate individual participant data. In HIV^–^ vaccinees, the Vδ2^+^ percentages increased after immunization and were higher than the Vδ2^+^ percentages in NS controls, although the difference did not reach statistical significance (*P* = 0.25, Wilcoxon-Mann-Whitney test adjusted for multiple comparisons). In HIV^+^ vaccinees, there was a small increase in the Vδ2^+^ percentages after immunization, which was not different from that of NS controls (*P* = 0.88). The percentages of CD3^+^ T cells staining Vδ2^+^ was higher in HIV^–^ vaccinees compared with HIV^+^ vaccinees before and after immunization (**C**).

Prior studies of PfSPZ Vaccine have shown an association between the levels of Vδ2^+^ gδ T cells prior to immunization and protection (5, 7, 19, 28). Although no statistically significant differences were identified, the following observations were made. In the HIV^–^ vaccinees, the median percentage of CD3^+^ T cells that were Vδ2^+^ (Figure 8A) was higher 2 weeks after the fourth dose compared with before immunization (4.41% versus 2.82%, *P* = 0.54) and higher than in the HIV^–^ controls (4.41% versus 1.35%, *P* = 0.25). In HIV^+^ vaccinees, the median percentage of CD3^+^ T cells that were Vδ2^+^ (Figure 8B) was higher 2 weeks after the fourth dose compared with before immunization (1.51% versus 0.75%, *P* = 0.70), but was not higher in vaccinees compared with the HIV^+^ controls (1.51% versus 1.90%, *P* = 0.88). The median percentage of CD3^+^ T cells that were Vδ2^+^ (Figure 8C) was higher in the HIV^–^ vaccinees compared with the HIV^+^ vaccinees before immunization (2.82% versus 0.75%) and after immunization (4.41% versus 1.51%, *P* = 0.59, Kruskal-Wallis).

## Discussion

The primary objective of this clinical trial was to determine whether PfSPZ Vaccine was safe and well tolerated in persons living with HIV. No solicited AEs were reported in either HIV^–^ participants or HIV^+^ participants with full viral suppression and CD4 counts of more than 500 cells/μL after 9.0 × 10^5^ PfSPZ of PfSPZ Vaccine administered on days 1, 3, 5, 7, and 29. Unsolicited AEs were infrequent in both HIV^+^ and HIV^–^ participants, with no differences between groups. Leukopenia and neutropenia were seen more often in the HIV^+^ vaccinees and controls in comparison with the HIV^–^ groups, but did not appear to be vaccine related. Our long-term goal of using PfSPZ Vaccine in MVPs to halt transmission and eliminate malaria in geographically focused areas (1, 29, 30) without having to screen for HIV status was supported by this trial.

A concern with live attenuated vaccines is breakthrough infection by the vaccine strain, which can occur if the live infectious agent is not completely attenuated and/or if the vaccinee’s immune system is required for complete attenuation and is functionally compromised. Disseminated varicella infection with vaccine strain has been reported in severely immunocompromised HIV^+^ individuals (31, 32). Bacille Calmette-Guérin (BCG) vaccination studies in HIV^+^ infants residing in Argentina and South Africa estimated disseminated infections occurred in approximately 1% (33–36) with up to 75% mortality. PfSPZ Vaccine has been administered to more than 2,000 vaccinees in previous trials with no vaccine breakthrough infections, and breakthrough infections were not expected in this trial because the attenuation of PfSPZ Vaccine is intrinsic to the parasite and not dependent on the recipient’s immune system (37).

Although safe, PfSPZ Vaccine was not protective in HIV^+^ participants. VE against homologous CHMI at 3 weeks after the last dose of vaccine was high, 80% (4 of 5), in HIV^–^ participants, but all 4 HIV^+^ participants developed Pf parasitemia. Reduced VE in HIV^+^ participants has been seen after immunization in other challenge or natural infection models (38–42), and the findings of our trial are consistent. Persistent, subtle immunologic deficits in HIV^+^ individuals despite long-term virologic suppression with antiretroviral agents are the most likely explanation. HIV^+^ individuals on fully suppressive antiretroviral therapy (ART) and with normal CD4^+^ T cell counts exhibit altered function and dysregulation of CD4^+^ T cells (43), Vγ9Vd2 T cells (44), and monocytes (45), and depressed protective T cell–dependent immune responses (43, 44). Infection of CD4^+^ T follicular helper cells (T_FH_), the major HIV reservoir in chronic asymptomatic infection, impairs B cell function in the germinal center, including maturation into plasma cells and long-lived memory B cells (46).

In this study, percentages of CD4^+^ T cells expressing IFN-γ, IL-2, TNF-α, or CD154 in response to in vitro PfSPZ stimulation were increased in both HIV^–^ and HIV^+^ vaccinees after the fourth immunization compared with the NS controls, with larger increases in HIV^–^ than HIV^+^ participants, although the differences in T cell responses between the HIV^–^ vaccinees (4 of 5 protected after CHMI) and the HIV^+^ vaccinees (0 of 4 protected after CHMI) were not significant. CD8^+^ T cell responses to PfSPZ were not detected 2 weeks after the fourth or fifth immunizations. We believe that the dramatic reduction in numbers of detectable PfSPZ-specific CD4^+^ T cells in PBMCs after the fifth dose and the inability to detect PfSPZ-specific CD8^+^ T cells in PBMCS were due to trafficking of the protective T cells to the liver (2), consistent with the observation that levels of PfSPZ-specific T cells in PBMCs are highest after the first dose (or priming doses) of PfSPZ Vaccine (7), and with data from mice immunized with radiation-attenuated rodent malaria SPZ, which show that the movement of specific CD8^+^ T cells, such as effector memory cells, to the liver occurs rapidly after challenge (47).

A role for Vγ9Vδ2 T cells in the induction of PfSPZ VE has been previously described (5, 7, 19, 28, 48). Early in HIV infection, peripheral blood Vδ1 T cell frequency is increased and Vδ2 T cell frequency is decreased, with a reversal of the Vδ1/Vδ2 ratio (which normally favors Vδ2) (44, 49), and the remaining Vδ2 T cells exhibit decreased antigen-induced expression of IFN-γ and TNF-α and decreased proliferative/cytotoxic capacity. These changes are incompletely reversed despite years of fully suppressive ART (44, 49). Although we did not find statistically significant differences, our results are consistent in that the percentages of CD3^+^ γδ T cells that were Vδ2^+^ were higher in the HIV^–^ vaccinees than HIV^+^ vaccinees before immunization and 2 weeks after the fourth immunization and that percentages increased after immunization in the HIV^–^ vaccinees while percentages in the HIV^+^ vaccinees remained indistinguishable from those of HIV^+^ controls after the fourth immunization. The T cell data generated from this trial suggest that Vγ9Vδ2 T cell function is an important component of the protective immune response. We are further exploring this using RNA-Seq, cellular indexing of transcriptomes and epitopes (CITE-Seq), and plasma proteomics.

Seroconversion to protective antibody responses and peak antibody responses with most licensed vaccines is depressed and not as long lived as in healthy HIV^–^ children and adults (50). Total (51) and neutralizing (51) antibody responses to PfCSP are similarly reduced in HIV^+^ children after receiving the RTS,S/AS01 vaccine. All vaccinees in this study had IgG and IgM antibody responses to PfCSP by ELISA (Figure 5, A and B) and IgG antibodies to PfSPZ by automated immunofluorescence assay (aIFA) (Figure 5C), which were nonsignificantly higher in HIV^–^ as compared with HIV^+^ vaccinees. However, the median serum dilution at which there was 80% inhibition in the functional aISI was significantly higher in the HIV^–^ vaccinees (21) as compared with the HIV^+^ vaccinees (11) (Figure 5D). It is possible that this functional antibody activity played a role in the VE in the HIV^–^ participants and the lack of VE in the HIV^+^ participants.

By using a systems immunology approach to characterize the binding and functional antibody responses to a panel of relevant Pf antigens, we found additional evidence that HIV^+^ individuals had weakened functional antibody responses. A striking functional antibody difference between HIV^+^ and HIV^–^ vaccinees was PfCSP antibody binding to FcR3B, an FcR highly expressed on the surface of neutrophils (52), and in vitro PfCSP ADNP activity. It has been suggested that ADNP activity is important for antisporozoite immunity (53), and the results of these 2 assays point to a defect in anti-PfCSP ADNP responses in HIV^+^ individuals. Further, our findings also point to decreased complement activation of anti-PfCSP antibodies in HIV^+^ vaccinees. The importance of complement-fixing antibodies in antisporozoite immunity has been demonstrated in RTS,S vaccinees (54) and in immunity to natural infection (55). Taken together, our findings suggest that deficits in functional anti-PfCSP humoral immunity distinguish HIV^+^ from HIV^–^ individuals. HIV^+^ individuals have a less responsive B cell compartment with compromised B cell memory and increased markers of B cell exhaustion (56, 57). This results in impaired responses to infections — in terms of both antibody level and quality (58). More work, such as antibody avidity and dissociation assessments, is needed to clarify the underlying mechanism driving this difference in vaccine response and potentially efficacy in order to find vaccination strategies that will protect both HIV^+^ and HIV^–^ individuals.

An interesting observation in this study was the number of Pf infections (7) identified in the HIV^+^ participants not attributable to PfNF54 — 2 during immunization (one of which either recrudesced during the 28-day period following CHMI or was replaced by a new infection) and 5 between days 28 and 56 after CHMI, following AL therapy (Figure 2). Six of the 7 infections were asymptomatic. In the HIV^–^ participants, parasitemia was only detected during the expected interval after CHMI (Figure 3), a finding consistent with 2 previous trials in HIV^–^ adults from the same study site (9, 11). Observational studies suggest both clinical malaria (59) and asymptomatic Pf parasitemia (60) may be more frequent or more severe in persons living with HIV, with higher levels of parasitemia (61) and correlated with lower CD4 counts (60, 62). Incidence of asymptomatic infection has not been shown to differ significantly, however, between HIV^+^ individuals with CD4 counts of more than 400 cells/μL and HIV^–^ individuals residing in the same community. Although the frequency of asymptomatic parasitemia in this study may have alternate explanations, such as different living conditions or areas of residence within Bagamoyo town that could increase exposure, these findings suggest increased susceptibility to infection in persons living with HIV, even with a fully suppressed HIV viral load and CD4 counts above 500 cells/μL.

### Limitations of the trial.

This trial provides evidence for the safety of PfSPZ Vaccine and PfSPZ Challenge in HIV^+^ individuals, with the caveat that all enrolled HIV^+^ participants were on antiretroviral treatment and had CD4^+^ T cell counts higher than 500/μL. Safety has yet to be established in those with depressed CD4^+^ T cell counts or suffering from opportunistic infections. Because this was a first trial that we know of in an immunocompromised study population, the number of participants was small, limiting the power to detect safety or immunology differences between groups. In addition, we have learned since performing this trial that clearance of parasitemia in all prospective vaccinees prior to the first dose is required for maximal vaccine-induced protection, even if the density of parasitemia is below the limit of detection by TBS, due to the immunosuppressive effects of parasitemia (63). Given the small sample size of the trial and the fact that all WT infections documented during immunization were in HIV^+^ participants, it is possible that an imbalance in subpatent (nondetectable by qPCR) parasitemias between the 2 groups contributed to the absence of protection seen in the HIV^+^ group. We also considered that the difference in age between the HIV^+^ (older) and HIV^–^ (younger) groups may have affected protection. However, a pooled analysis of CHMI outcomes in 80 HIV^–^ vaccinees 18 to 48 years of age from 4 trials receiving 9.0 × 10^5^ PfSPZ of PfSPZ Vaccine on days 1, 8, and 29 showed no significant difference in age between participants who did or did not develop parasitemia by qPCR by day 28 (our unpublished observations).

We achieved the primary goal of this clinical trial, establishing that PfSPZ Vaccine was well tolerated and safe in a small number of HIV^+^ individuals. Expanded testing of PfSPZ Vaccine in larger numbers of HIV^+^ participants, including those with lower CD4^+^ T cell counts or viremia or others with immunosuppressed status, is merited. However, to make PfSPZ vaccines most useful, we must determine how to overcome the lack of VE of PfSPZ Vaccine in the HIV^+^ participants. One approach will be to assess different doses or dosing regimens of PfSPZ Vaccine, including our downselected regimen of 9.0 × 10^5^ PfSPZ on days 1, 8, and 29. Another will be to include the institution of presumptive treatment for malaria prior to the first vaccine dose to reduce the immunosuppressive effects of latent parasitemia. A third will be to move to more potent PfSPZ vaccines, such as PfSPZ-CVac (CQ) (64, 65), or genetically attenuated late arresting replication competent (LARC) PfSPZ vaccines, which have been manufactured and will enter clinical trials in 2024 (66).

## Methods

### Study design and population

This single-center, randomized, double-blind, placebo-controlled trial was conducted in the Bagamoyo Clinical Trials Unit, IHI, between February 2018 and August 2018. Healthy HIV^–^ and HIV^+^ males and females ages 18 to 45 years were enrolled after they provided informed consent, successfully completed a test of study understanding, and met prespecified inclusion and exclusion criteria (ClinicalTrials.gov NCT03420053) (Supplemental Table 11 and Supplemental Table 12). For HIV^+^ participants, this included undetectable HIV viral load by PCR, CD4 of more than 500 cells/μL, and a stable antiretroviral regimen for at least 3 months.

### Investigational products

Sanaria PfSPZ Vaccine comprises radiation-attenuated, aseptic, purified, vialed, cryopreserved PfSPZ stored in liquid nitrogen vapor phase (LNVP) at –150 to –196°C. Preparation of investigational productions (IP) in 0.5 mL of diluent (human serum albumen in PBS) was done under the supervision of the study pharmacist. PfSPZ Vaccine or NS (0.5 mL) was administered by DVI through a 25-gauge needle. Sanaria PfSPZ Challenge (NF54) was used for CHMI. PfSPZ Challenge is identical to PfSPZ Vaccine, except it is not radiation attenuated and is infectious. Handling and administration were similar to that of PfSPZ Vaccine. NS was the placebo, as it is indistinguishable from the vaccine, can be administered intravenously, and is virtually free of adverse effects, providing a strict comparator for the assessments of safety.

### Care for HIV+ participants

Maintaining the standard of care for HIV^+^ adults provided by national Care and Treatment Centers (CTC) was prioritized to maximize safety for the HIV^+^ study participants. The study team coordinated care with CTC staff throughout the course of the study. All HIV^+^ participants remained on ART during the trial. Assessments for safety included clinical evaluation with WHO staging, assessments of medication adherence, and safety laboratory assessments, including CD4 counts and HIV viral loads (Supplemental Table 13). Clinical and laboratory evaluation at the CTCs were maintained and encouraged; participants could be referred for care to the CTC providing their HIV-related care, should there be indications of clinical failure (a new or recurrent clinical event indicating severe immunodeficiency after 6 months of effective treatment), immunological failure (CD4 count <250 cells/mm^3^ following clinical failure or persistent CD4 levels <100 cells/mm^3^), or virological failure (HIV viral load above 1,000 copies/ml based on 2 consecutive viral load measurements within 3 to 6 months, with medication adherence support following the first viral load test) (67).

### Randomization and intervention

Twenty-one adult participants were allocated into 3 groups based upon HIV status. Group 1 consisted of 9 non–HIV-infected participants randomized to receive 9.0 × 10^5^ PfSPZ of PfSPZ Vaccine (*n* = 6) or NS control (*n* = 3) on days 1, 3, 5, 7, and 29 by DVI. Following a safety assessment 7 days after the fourth dose in group 1, a sentinel safety group (group 2a) of HIV^+^ participants (*n* = 3) received 4.5 × 10^5^ PfSPZ of PfSPZ Vaccine on days 1, 3, 5, 7, and 29 by DVI. Following a safety assessment 7 days after the fourth dose in group 2a, 9 HIV^+^ participants allocated to group 2b were randomized to receive 9.0 × 10^5^ PfSPZ of PfSPZ Vaccine (*n* = 6) or NS (*n* = 3) on days 1, 3, 5, 7, and 29 by DVI. Randomization was by a computer-generated list without restriction, with each participant entered sequentially to the list as they were enrolled. Only the lead pharmacist had access to this list. For groups 1 and 2b, the participants and investigators were blinded to treatment assignment.

### AE assessment

Solicited AEs following immunization were recorded utilizing prespecified criteria (Supplemental Table 4) beginning after the first immunization until 2 days (local) or 7 days (systemic) after the fourth immunization and for 2 (local) or 7 (systemic) days after the fifth immunization. Solicited AEs following administration of PfSPZ Challenge were recorded utilizing prespecified criteria for 2 (local) or 5 (systemic) days after CHMI (Supplemental Table 4). Subsequent assessment of an expanded set of solicited systemic AEs representative of symptomatic Pf infection continued until a participant was diagnosed and completed treatment for malaria or until day 28 after CHMI (Supplemental Table 4). Unsolicited AEs were assessed by an open-ended questioning and recorded from day 1 to day 14 after each immunization or series of immunizations and to day 28 after administration of PfSPZ Challenge for CHMI. All AEs were assessed for severity and relatedness to PfSPZ Vaccine or PfSPZ Challenge administration; these assessments were blinded for groups 1 and 2b. Laboratory abnormalities, including those involving hematology, ALT, creatinine, CD4, and quantitative HIV nucleic acid testing (Cepheid Xpert HIV-1), were assessed for changes from baseline, severity, and relatedness (Supplemental Table 13).

### VE

VE was assessed by homologous CHMI with 3.2 × 10^3^ PfSPZ of PfSPZ Challenge administered by DVI 3 weeks after the last immunization for participants in groups 1 and 2b. Participants were admitted to the facility 7 days after receiving PfSPZ Challenge (day 8), and inpatient observations were performed continuously until either (a) patients were diagnosed with malaria, treated, and confirmed to be malaria free (documented 2 negative daily blood smears) or (b) at 21 days (day 22) after receiving PfSPZ Challenge, if no parasitemia had been detected. TBSs were collected and read twice daily from day 8 to day 15 and then once daily until discharge on day 22. All participants who received PfSPZ Challenge were seen for follow-up 28 days after CHMI for safety assessment and TBS/qPCR and presumptive treatment with antimalarials (see below). Participants who were treated for documented parasitemia through day 29 and who were taking efavirenz or nevirapine for ART had an additional sample drawn for blood smear and PCR on day 43. The final clinical review was scheduled at a study visit on day 57.

### Detection of Pf parasites

Participants were assessed for parasitemia by TBS and qPCR at screening, prior to the first immunization and prior to CHMI. AFter CHMI, TBSs were prepared and read on days 6 and 7, twice daily on days 8 to 14, and daily on days 15 to 20 or until positive, with daily qPCR samples obtained over the same interval. Follow-up TBSs and qPCR were assessed on day 28 after CHMI in all participants and again on day 42 after CHMI in any HIV^+^ participant taking efavirenz or nevirapine who was treated for parasitemia in the 28 days after CHMI.

Slide preparation and reading for TBSs were performed as described (68). In brief, 10 μL of blood collected in EDTA was placed on a 10 mm by 20 mm rectangle on a glass slide, dried, and stained. For asymptomatic individuals, approximately 0.5 μL of blood was assessed. For symptomatic individuals, approximately 2.0 μL of blood was assessed. Two asexual erythrocytic stage Pf parasites had to be identified for a slide to be considered positive. All positive TBSs were confirmed by qPCR prior to the initiation of treatment. TBSs were continued after initiation of treatment until 2 consecutive smears were negative.

Parasites were quantified by the PlasQ assay, a multiplex qPCR assay targeting the pan-*Plasmodium* spp. 18S rDNA (69) and the high copy number Pf-specific varATS region (70), which has an overall lower limit of detection of 50 parasites/mL (71), using a sample volume of 180 μL. A single positive result was considered positive for infection with Pf. After the start of CHMI, the time of first blood sample positivity by qPCR was used to determine infection status and calculation of prepatent period. All samples were analyzed by qPCR in real time as they were continuously collected from the participants.

Parasite genotyping was applied to selected samples when necessary to distinguish NF54 from naturally acquired Pf infection. Genotyping was performed by the Swiss Tropical Public Health Institute using published methods (72), targeting the sequences for merozoite surface protein (MSP) 1 and 2, glutamate-rich protein (GLURP), and microsatellites PfPK2, TA40, TA60, and TA81 to establish identification.

### Treatment of Pf infections

Symptomatic and asymptomatic *Plasmodium* sp. infections diagnosed or confirmed by qPCR in participants prior to the CHMI were treated according to national guidelines with AL (80 mg artemether/480 mg lumefantrine twice a day for 3 days) under directly observed therapy (DOT). All participants with positive TBSs in the 28-day interval following CHMI were treated with AL within 24 hours of first positive TBS confirmed by qPCR. Participants remaining TBS negative were presumptively treated with AL on day 28, regardless of qPCR findings.

### Immunology assays

Samples for analysis of humoral and cellular immune responses and transcriptomics were obtained prior to each immunization and prior to CHMI and at specific times after immunizations and after CHMI.

#### Standard antibody assays.

Sera were separated and frozen at –80°C within 4 hours of collection. IgG and IgM antibodies to PfCSP and IgG antibodies to MSP5 were assessed by ELISA and IgG antibodies to PfSPZ by aIFA, as described (64, 65). Functional activity was assessed by the ISI assay (64, 65). Definitions for a positive response were taken relative to the pre–dose 1 measurement. For ELISA, samples were considered positive if the difference between the postimmunization OD 1.0 and the preimmunization OD 1.0 (net OD 1.0) was 50 or more and the ratio of the postimmunization OD 1.0 to preimmunization OD 1.0 (ratio) was 3.0 or more. For aIFAs, participants with a net reciprocal serum dilution for 2 Å~ 105 arbitrary fluorescence units (AFUs) of 150 or more and a ratio reciprocal serum dilution of 3.0 or more were considered positive. For aISI, participants with a net reciprocal serum dilution for 80% inhibition of 10 or more in the ISI assay and a ratio reciprocal serum dilution for 80% inhibition of 3.0 or more in the ISI were considered positive.

#### Systems serology assays.

Recombinant PfCSP, PfSSP2/PfTRAP, PfAMA1, PfEXP1, PfLSA1, and PfEBA175 were used. Antigen-specific isotype level and FcR binding were measured by a multiplex Luminex assay, as previously described (73). The ADNP assay was performed with neutrophils isolated from 2 separate human donors, and the antibody-dependent cell phagocytosis (ADCP) assay was performed with THP-1 cells in technical duplicate, both as previously described (73). ADCD was performed with a Luminex bead array assay measuring C3 deposition triggered by antigen-specific polyclonal sera adapted from previously published work (74).

#### T cell analyses.

T cell responses in cryopreserved PBMCs were assessed by multiparameter flow cytometry as described previously for CD4^+^ and CD8^+^ (5). After thawing, PBMCs were rested for 8 hours, then stimulated for 17 hours with PfSPZ Vaccine diluent (PBS with 1% human serum albumin; CSL Behring) or 1.5 × 10^5^ viable, irradiated, aseptic, purified, cryopreserved PfSPZ from a single production lot. For the last 5 hours of the stimulation, 10 μg/ml brefeldin A (BD Biosciences) was added to the culture. After stimulation, cells were stained as previously described (75). Briefly, cells were washed and stained with viability dye for 20 minutes at room temperature, followed by a 20-minute surface staining at room temperature for the markers CD4, CD8, CD14, CD20, CD38, CD45RA, CD56, TCR Vα-7.2, CD161, TCR-γδ, TCR-Vδ1, TCR-Vδ2, TCR-Vγ9, or CXCR6. Cells were washed, fixed, and permeabilized using the Cytofix/Cytoperm kit (BD Biosciences) and were stained intracellularly for CD3, IFN-γ, IL-2, TNF-α, perforin, and Ki-67. On completion of staining, cells were collected on a BD FACSymphony Flow Cytometer. Samples were analyzed using FlowJo, version 10.6.1 (FlowJo LLC). Cytokine-positive cells were determined after gating on nonnaive T cells. All antigen-specific cytokine frequencies were reported after background subtraction of identical gates from the same sample incubated with negative control stimulation (human serum albumin).

Ex vivo measurements of Vd2^+^ and Vd2^–^ gδ T cells were performed using PBMCs. Briefly, cryopreserved PBMCs were thawed and stained with Aqua Viability Dye (Invitrogen) for 15 minutes. Cells were then washed and surface stained with a cocktail of antibodies for anti-CD3 Alexa Fluor 700, CD4-BV421, CD8 allophycocyanin-H7, gdTCR-PE, CD27–PE–Cy7, CD45RO-PECF594, and Vδ2-FITC and incubated for 20 minutes (Supplemental Figure 7). All antibodies were purchased from BD Biosciences. Cells were washed and acquired on an LSR II flow cytometer equipped with a blue, red, and violet laser. Subsequent analysis of flow cytometry data was done using FlowJo (version 10.5.3).

### Statistics

Small group sizes were selected to balance the chance to detect any possible untoward reactions against the desire to limit the number of participants involved for safety purposes. All participants who received at least 1 dose of IP were included in the safety analysis; all participants who participated in CHMI were included in the efficacy analysis except 1 HIV^+^ participant who was found to have a non-NF54 parasite infection. Categorical variables were summarized using absolute (*n*) and relative (%) frequencies. Continuous variables were summarized using mean and SD, median, and range. Comparisons of categorical variables between groups were analyzed using Barnard’s 2-sided exact unconditional test; comparisons of continuous variables were analyzed using the Mann-Whitney 2-sided test. VE was estimated as 1– (attack rate in vaccine participants/attack rate in NS control participants) based upon parasitemia detected by qPCR. For purposes of VE calculations, the HIV^–^ and HIV^+^ participants receiving placebo were to be combined, assuming infection rates would be similar in both.

The immune responses of study participants in each group were compared with the immune responses of the corresponding placebo groups. For standard antibody assays, differences between vaccinees and controls were analyzed using the Mann-Whitney test for net OD 1.0 and OD 1.0 ratios. The Holm-Bonferroni method was used to calculate adjusted *P* values. For systems serology analyses, univariate and multivariate statistical analyses were performed in R (version 4.0.0) or Python (version 3.9.1). Prior to analysis, Luminex and ADCD data were log_10_ transformed, and all data were centered and scaled. For univariate analysis, significance was determined by Mann-Whitney *U* test. For feature selection, the systemseRology R package (version 1.0) (https://github.com/LoosC/systemsseRology) was used. Elastic-net parameters were optimized using tuneLength of 10 and leave-one-out cross validation. PCA of the elastic-net selected features was performed with the sklearn package (version 1.1.2). Cocorrelation network analysis was performed in Cytoscape (version 3.8).

For T cell analyses, the differences in the percentages of CD4^+^ T cells expressing cytokines or expressing markers of activation were analyzed using the Kruskal-Wallis test and adjusted for multiple comparisons using the Holm-Bonferroni method to calculate an adjusted *P* value. Any *P* value of less than 0.05 was considered significant.

### Study approval

This clinical trial (BSPZV3a) is registered at ClinicalTrials.gov (NCT03420053). Following successful completion of a written test of understanding, all study participants provided written, informed consent prior to participation in the trial. This trial was approved by the IRBs of the IHI and the National Institute for Medical Research (NIMR), both in Tanzania, and was conducted in compliance with the Declaration of Helsinki and Good Clinical Practice guidelines. Results are presented following the CONSORT reporting guidelines (76).

### Data availability

The study protocol and all data for this trial held by Sanaria are available through the ImmPort data repository (Study SDY1909; www.ImmPort.org). Values for all data points in graphs are reported in the Supporting Data Values file.

## Author contributions

SJ was the principal investigator (PI) and FM the lead clinician of the clinical trial. SJ, TLR, SA, and SLH designed the trial. Conduct of the trial was supported by MQ, MR, AT, GN, BMB, LM, KK, BSS, ERJ, and PFB. BKLS, TM, YA, and ERJ provided the study product. TS, M Mpina, and CD managed the clinical laboratory, including performance or TBS and diagnostic PCR. TAM, PR, and LWPC were responsible for data management and analysis. NK, JDH, GA, YZ, and M Mendu performed and analyzed the humoral immunology studies. IZ, PED, PAS, and RS performed and analyzed cellular immunology. LWPC, SJ, and SLH wrote the manuscript with contributions from all authors in the writing or review stages. SJ and LWPC are co–first authors, with SJ listed first as the study PI.

## Supplementary Material

Supplemental data

ICMJE disclosure forms

Supporting data values

## Version 1 01/09/2024

In-Press Preview

## Version 2 03/15/2024

Electronic publication
