# Supplementary material for: Safety and protective efficacy of PfSPZ Vaccine administered to HIV-negative and -positive Tanzanian adults
Source: J Clin Invest. 2024 Jan 9;134(6):e169060. doi: 10.1172/JCI169060 (PMC10940097; doi:10.1172/JCI169060)

SUPPLEMENTAL DATA

Table S1: Participant demographics.

	HIV-		HIV+		
	$9.0x10^5$ PfSPZ N=6	NS N=3	$4.5x10^5$ PfSPZ N=3	$9.0x10^5$ PfSPZ N=6	NS N=3
**Age - median, range**	25.5 (18 - 33)	24 (21 – 25)	33 (30 – 43)	41.5 (30 – 43)	38 (28 – 40)
**Gender**					
**male**	4	2	1	3	3
**female**	2	1	2	3	0
**Weight (kg), median**	57.5 (48,61)	62 (53,64)	65 (50,70)	55 (48,82)	63 (58,75)
**male**	60 (56,64)	63 (62,64)	65	58 (48,82)	63 (58,75)
**female**	51.5 (48,55)	53	60 (50,70)	52 (51,65)	
**BMI, median**	20.5 (19,21)	21 (20,21)	23 (22,29)	21.5 (19,30)	22 (19,29)
**male**	20.5 (19,21)	20.5 (20,21)	22	23 (19,27)	22 (19,29)
**female**	20.5 (20,21)	21	26 (23,29)	20 (19,30)	

Table S2: Individual demographics and HIV history for the HIV Positive Participants.

	Sex	Age (years)	Months since diagnosis (at enrollment)	CD4 nadir* (cells/µL)	CD4 at enrollment (cells/µL)	Antiretroviral regimen at enrollment	# months on current ARV
J	male	43	104	290	1009	tenofovir + emtricitabine + efavirenz	49
K	female	33	74	355	951	tenofovir + lamivudine + efavirenz	48
L	female	30	15	547	1021	tenofovir + lamivudine + efavirenz	15
M	male	38	16	509	571	zidovudine + lamivudine + efavirenz	16
N	male	43	138	525	680	zidovudine + lamivudine + nevirapine	138
O	male	42	91	444	704	tenofovir + lamivudine + efavirenz	88
P	male	28	44	481	754	tenofovir + lamivudine + efavirenz	9
Q	male	43	16	628	623	tenofovir + lamivudine + efavirenz	15
R	female	30	10	801	1262	tenofovir + lamivudine + efavirenz	10
S	female	41	98	133	701	zidovudine + lamivudine + nevirapine	74
T	male	40	121	207	674	tenofovir + lamivudine + efavirenz	64
U	female	35	127	495	509	zidovudine + lamivudine + nevirapine	84

*Lowest CD4 count on record with the Clinical Treatment Center, may not reflect true nadir CD4

Table S3: Reasons for screening failure. Twenty-eight individuals (1 HIV- and 27 HIV+) did not meet the inclusion/exclusion criteria for enrolment.

**Inclusion/exclusion criterion**	**Number not meeting criterion***
BMI <18 or >30 kg/m^2	2
WHO HIV stage $\geq$2	2
CD4 T cell count < 500 cells/µL	9
Positive QuantiFERON-TB test	9
Positive for hepatitis B or hepatitis C	1
Medical, social condition or occupational reason that, in the judgment of the investigator, is a contraindication to protocol participation, may impair the volunteerâ€™s ability to give informed consent or effectively participate in the study, may significantly increase the risk to the volunteer because of participation in the study or may impair interpretation of the study data	7

*Individuals screened could fail to meet more than 1 criteria

Table S4: Pf genotyping. To determine if late positive samples represented recrudescence or new infections, the late positive samples and the preceding positive samples for selected study participants were submitted to the Swiss Tropical Public Health Institute for genotyping. NF54, the strain used for CHMI, was confirmed for 2 participants (R, T). One participant (N) had a naturally acquired strain during the CHMI observation period. In 2 participants (M, S) infections could not be confirmed by a lower sensitivity qPCR and in a 3rd (P) genotyping was unsuccessful.

Patent ID	Visit ID	Original Parasite Density by qPCR	QC (qPCR Cq for Pf)	MSP2			MSP1				GLURP		Microsatellites				Combined outcome (sequential typing)
				FC27	3D7	Outcome MSP2	Mad20	K1	RO33	Outcome MSP1	GLURP	Outcome Glurp	PfPK2	TA40	TA60	TA81	
NF54 reference				neg	257.37		neg	240.48	neg		872.52; 880.00		247.30	270.35	325.09	182.19	
M	V4+2	0.14	neg	-	-	-	-	-	-	-	-	-	-	-	-	-	-
	V5 pre	0	neg	-	-	-	-	-	-	-	-	-	-	-	-	-	-
	CH+18	0.08	neg	-	-	-	-	-	-	-	-	-	-	-	-	-	-
	CH+56	0.04	neg	-	-	-	-	-	-	-	-	-	-	-	-	-	-
N	V5 pre	1040.71	*	-	-	-	-	-	-	-	-	-	-	-	-	-	-
	CH+20	234.41	27.9	399.91	neg	other	neg	222.97	neg	other	930.87; 938.76	other	267.81	261.45	315.79	188.07	other
P	CH+16	9.59	*	-	-	-	-	-	-	-	-	-	-	-	-	-	-
	CH+56	0.06	35.5	-	-	-	-	-	-	-	-	-	-	-	-	-	-
R	CH+16	32.56	28.7	neg	257.41	NF54	neg	240.49	neg	NF54	872.74; 880.47	NF54	247.27	270.35	325.13	182.17	NF54
	CH+56	0.1	neg	-	-	-	-	-	-	-	-	-	-	-	-	-	-
S	CH pre	0.12	neg	-	-	-	-	-	-	-	-	-	-	-	-	-	-
	CH+56	0.05	neg	-	-	-	-	-	-	-	-	-	-	-	-	-	-
T	CH+12.5	9.99	32	neg	neg	undetermined	neg	240.49	neg	NF54	872.63; 880.47	NF54	247.24	270.33	325.11	182.22	NF54
	CH+13		31.1	neg	257.31	NF54	neg	240.53	neg	NF54	872.20; 880.12	NF54	247.29	270.44	325.05	182.11	NF54
	CH+56	15266.1	23.6	neg	292.64	other	neg	neg	217	other	**872.83; 880.29;** 940.32; 947.61	NF54	238.42; 244.31	270.35	321.91	182.17	other

*sample not received for genotyping

V4+2 – 2 days after 4th dose; V5 pre – immediately prior to 5th dose; CH pre – immediately prior to CHMI; CH+XX – days after CHMI

Table S5: Solicited local and systemic adverse events – list of specific signs and symptoms included and the intervals post immunization or CHMI.

<table>
<tr><th colspan="2">Attribution of Local Symptoms and Signs (Solicited AEs)</th><th>PfSPZ Vaccine</th><th>PfSPZ-CHMI</th></tr>
<tr><td>At injection site</td><td>• Pain
• Pruritus
• Swelling
• Tenderness
• Erythema
• Induration
• Bruising</td><td>V
Through
V+2</td><td>CH
Through
CH+2</td></tr>
<tr><th colspan="2">Attribution of Systemic Symptoms and Signs (Solicited AEs)</th><th>PfSPZ Vaccine</th><th>PfSPZ-CHMI</th></tr>
<tr><td>Symptoms and Signs attributed to:

Post DVI and SPZ development in the liver</td><td>• Fever
• Allergic reaction (rash, urticaria, pruritis, edema)
• Headache
• Subjective Fever**
• Fatigue
• Malaise
• Chills
• Myalgia
• Arthralgia</td><td>▪ V through V+7</td><td>▪ CH through CH+5</td></tr>
<tr><td>Symptoms and Signs attributed to:

Post-Challenge Parasitemia (CHMI)</td><td>• Fever
• Allergic reaction (rash, urticaria, pruritis, edema)
• Headache
• Subjective Fever**
• Fatigue
• Malaise
• Chills
• Myalgia
• Arthralgia
• Dizziness
• Rigors
• Sweats
• Cough
• Nausea
• Vomiting
• Abdominal pain
• Diarrhea
• Chest pain
• Palpitations
• Shortness of breath</td><td>▪ NA</td><td>CH+6 through CH+28</td></tr>
</table>

*Toxicity grading for severity of these signs and symptoms are described in section **Error! Reference source not found..**

**Perceived by the volunteer

Table S6: Unsolicited adverse events after immunization. All events were considered unrelated to immunization. Grade 2 events noted by *, all other events were grade 1.

**Adverse event**	**HIV-**		**HIV+**	
	**PfSPZ Vaccine N=6**	**NS N=3**	**PfSPZ Vaccine N=9**	**NS N=3**
Abdominal pain	1	1	0	0
Diarrhea	0	0	1*	0
Vaginal bleeding/ Incomplete abortion	1*	0	0	0
Superfical burn	1	0	0	0
Contact dermatitis	1	0	0	0

N=Number of participants in the safety population

Table S7: Number and Percentage of Participants With Abnormal Safety Laboratory Results by Treatment Group.

Laboratory Parameter	Severity	Group 1 (HIV seronegative)		Group 2a (HIV seropositive)	Group 2b (HIV seropositive)	
		$9x10^5$ N=6	Placebo N=3	$4.5x10^5$ N=3	$9x10^5$ N=6	Placebo N=3
Any lab abnormality (from list below)	Grade 1	2 (33%)	1 (33%)	2 (67%)	3 (50%)	3 (100%)
	Grade 2	1 (17%)	0	1 (33%)	2 (33%)	0
	Grade 3	0	0	0	1 (17%)	0
Hemoglobin (decreased)	Grade 1	1 (17%)	1 (33%)	0	2 (33%)	2 (67%)
	Grade 2	1 (17%)	0	1 (33%)	0	
WBC (decreased)	Grade 1	1 (17%)	1 (33%)	2 (67%)	0	2 (67%)
	Grade 2	0	0	0	3 (50%)	0
Neutrophil Count (decreased)	Grade 1	1 (17%)	0	0	1 (17%)	2 (67%)
	Grade 2	0	0	0	0	0
	Grade 3	0	0	0	1 (17%)	0
Lymphocyte Count (decreased)	Grade 1	1 (17%)	0	0	2 (33%)	0
Eosinophil Count (increased)	Grade 1	1 (17%)	1 (33%)	0	1 (17%)	0
Platelets (decreased)	Grade 1	0	0	0	0	1 (33%)
ALT (increased)	Grade 1	1 (17%)	0	0	2 (33%)	0
AST (increased)	Grade 1	0	0	0	0	0
Creatinine (increased)	Grade 1	0	1 (33%)	0	1 (17%)	1 (33%)

N=Number of participants in the Safety population

Table S8: Number and Percentage of Volunteers Experiencing Solicited Adverse Events During CHMI by Sign/Symptom and Group. All solicited AE are considered related to PfSPZ Challenge (CHMI days 1-6) or Pf parasitemia (CHMI days 7-29)

Sign/Symptom	Period of Assessment	Group 1 (HIV seronegative)		Group 2b (HIV seropositive)	
		$9x10^5$ N=5	Placebo N=3	$9x10^5$ N=5	Placebo N=3
**Any Sign/Symptom**	Days 1 - 6 (CH to CH+5)	0	0	1 (20%)	0
	Days 7 to 29 (CH+6 to CH+28)	0	1 (33.3%)	3 (60%)	2 (66.6%)
**Any Local Sign/Symptom**	Days 1-3 (CH to CH+2)	0	0	0	0
Tenderness	Days 1-3 (CH to CH+2)	0	0	0	0
Induration	Days 1-3 (CH to CH+2)	0	0	0	0
Erythema	Days 1-3 (CH to CH+2)	0	0	0	0
Swelling	Days 1-3 (CH to CH+2)	0	0	0	0
Bruising	Days 1-3 (CH to CH+2)	0	0	0	0
Pain*	Days 1-3 (CH to CH+2)				
Pruritus	Days 1-3 (CH to CH+2)	0	0	0	0
**Any Systemic Sign/Symptom**	Days 1 - 6 (CH to CH+5)	0	0	1 (20%)	0
	Days 7 to 29 (CH+6 to CH+28)	0	1 (33.3%)	3 (60%)	2 (66.6%)
Increased Oral Temperature	Days 1 - 6 (CH to CH+5)	0	0	0	0
	Days 7 to 29 (CH+6 to CH+28)	0	0	0	0
Systemic rash, urticaria, edema, pruritis	Days 1 - 6 (CH to CH+5)	0	0	0	0
	Days 7 to 29 (CH+6 to CH+28)	0	0	0	0
Subjective Fever/Fever	Days 1 - 6 (CH to CH+5)	0	0	0	0
	Days 7 to 29 (CH+6 to CH+28)	0	1 (33.3%)	1 (20%)	1 (33.3%)
Chills	Days 1 - 6 (CH to CH+5)	0	0	0	0
	Days 7 to 29 (CH+6 to CH+28)	0	1 (33.3%)	0	0
Headache	Days 1 - 6 (CH to CH+5)	0	0	1 (20%)	0
	Days 7 to 29 (CH+6 to CH+28)	0	1 (33.3%)	3 (60%)	2 (66.6%)
Fatigue	Days 1 - 6 (CH to CH+5)	0	0	1 (20%)	0
	Days 7 to 29 (CH+6 to CH+28)	0	1 (33.3%)	0	1 (33.3%)
Malaise	Days 1 - 6 (CH to CH+5)	0	0	0	0
	Days 7 to 29 (CH+6 to CH+28)	0	1 (33.3%)	1 (20%)	0
Myalgia	Days 1 - 6 (CH to CH+5)	0	0	0	0
	Days 7 to 29 (CH+6 to CH+28)	0	0	0	0
Arthralgia	Days 1 - 6 (CH to CH+5)	0	0	1 (20%)	0
	Days 7 to 29 (CH+6 to CH+28)	0	1 (33.3%)	0	1 (33.3%)

Sign/Symptom	Period of Assessment	Group 1 (HIV seronegative)		Group 2b (HIV seropositive)	
		$9x10^5$ N=5	Placebo N=3	$9x10^5$ N=5	Placebo N=3
Abdominal Pain	Days 7 to 29 (CH+6 to CH+28)	0	0	0	0
Chest Pain	Days 7 to 29 (CH+6 to CH+28)	0	0	0	0
Cough	Days 7 to 29 (CH+6 to CH+28)	0	0	0	0
Diarrhea	Days 7 to 29 (CH+6 to CH+28)	0	0	0	0
Dizziness	Days 7 to 29 (CH+6 to CH+28)	0	0	0	0
Nausea	Days 7 to 29 (CH+6 to CH+28)	1 (20%)	0	0	0
Palpitations	Days 7 to 29 (CH+6 to CH+28)	0	0	0	0
Rigors	Days 7 to 29 (CH+6 to CH+28)	0	0	0	0
Shortness of Breath	Days 7 to 29 (CH+6 to CH+28)	0	0	0	0
Sweats	Days 7 to 29 (CH+6 to CH+28)	0	0	0	0
Vomiting	Days 7 to 29 (CH+6 to CH+28)	1 (20%)	0	0	0

N = Number of subjects participating in CHMI (Efficacy Population).

*Pain is specified as a solicited local adverse event per protocol but was not included in the CRFs. Pain is consequently not captured as an independent event but was recorded as part of tenderness.

Table S9: Immunological data for all volunteers who received at least one dose of PfSPZ Vaccine, as measured by PfCSP ELISA (panel A), PfMSP5 ELISA (panel A), aIFA (panel B), and ISI (panel B) assays. All out-of-range values, negatives and zeroes are reported as 1**.**

A.

Grp	Volunteer ID	HIV+/-	Infection	SPZ/Inj	ELISA											
					PfCSP IgG OD1.0				PfCSP IgM OD1.0				IgG Antibodies against PfMSP5			
					Pre-V1	V5+14	Net V5+14	Ratio V5+14	Pre-V1	V5+14	Net V5+14	Ratio V5+14	Pre-V1	V5+14	Net V5+14	Ratio V5+14
1	B	-	No	900,000	158	11,554	11,396	72.1	81	9,607	9,526	118.6	1,042	1,191	149	1.14
1	C	-	No	900,000	22	3,509	3,487	158.5	1	12,200	12,199	12200.0	375	328	-47	0.87
1	G	-	No	900,000	95	34,911	34,816	366.5	214	30,740	30,526	143.6	3,031	2,516	-515	0.83
1	I	-	No	900,000	13	10,650	10,637	818.2	1	6,268	6,267	6268.0	433	559	126	1.29
1	*F*[1]	-	N/A	900,000	193	26,655	26,462	137.1	233	161,214	160,981	691.9	1,919	2,891	972	1.51
1	D	-	Yes	900,000	70	6,152	6,082	86.9	100	9,488	9,388	94.9	477	299	-178	0.63
1	E	-	Yes	Placebo	70	72	2	1.0	142	149	7	1.0	680	509	-171	0.75
1	H	-	Yes	Placebo	32	23	1	1.0	272	75	1	1.0	472	337	-135	0.71
1	A	-	Yes	Placebo	17	26	9	0.5	1	111	110	111.0	376	326	-50	0.87
2a	J	+	N/A	450,000	16	1,333	1,317	82.3	37	54,611	54,574	1476.0	455	1,022	567	2.25
2a	L	+	N/A	450,000	2,849	25,807	22,958	8.1	79	29,448	29,369	372.8	516	987	471	1.91
2a	K	+	N/A	450,000	107	7,005	6,898	64.5	344	6,460	6,116	18.8	534	876	342	1.64
2b	R	+	Yes	900,000	15	7,731	7,716	514.4	197	218,700	218,503	1110.2	463	723	260	1.56
2b	Q	+	Yes	900,000	76	3,774	3,698	48.7	197	13,047	12,850	66.2	644	549	-95	0.85
2b	O	+	Yes	900,000	99	6,469	6,370	64.3	213	18,296	18,083	85.9	259	232	-27	0.90
2b	*U*[2]	+	Yes	900,000	64	6,262	6,198	96.8	195	26,420	26,225	135.5	394	306	-88	0.78
2b	*N*[3]	+	Yes	900,000	85	3,665	3,580	42.1	1	4,303	4,302	4303.0	494	1,994	1,500	4.04
2b	*S*[4]	+	N/A	900,000	109	10,680	10,571	97.0	1	10,080	10,079	10080.0	2,428	1,585	-843	0.65
2b	P	+	Yes	Placebo	1	22	21	21.0	24	37	36	1.5	173	160	-13	0.92
2b	T	+	Yes	Placebo	113	1	1	1.0	1	1	1	1.0	266	275	9	1.03
2b	M	+	Yes[5]	Placebo	480	347	1	1.0	3,306	602	1	1.0	2506	2,227	-279	0.89

[1]did not receive V5, no CHMI
[2]did not receive V3
[3]did not receive V5
[4]did not receive CHMI
[5]qPCR +, TBS -

B.

Grp	Volunteer ID	HIV+/-	Infection	SPZ/Inj	aIFA AFU 2.0×10^5				ISI Reciprocal serum dilution for 80% inhibition			
					Pre-V1	V5+14	Net V5+14	Ratio V5+14	Pre-V1	V5+14	Net V5+14	Ratio V5+14
1	B	-	No	900,000	1	2660	2659	2660.2	1	30	29	30.1
1	C	-	No	900,000	15	950	935	63.1	1	16	15	15.6
1	G	-	No	900,000	1	5379	5378	5379.2	1	28	27	27.8
1	I	-	No	900,000	1	1249	1248	1249.2	1	22	21	22.3
1	*F*[1]	-	N/A	900,000	1	4561	4560	4560.6	1	15	14	15.2
1	D	-	Yes	900,000	1	699	698	699.1	1	19	18	18.8
1	E	-	Yes	Placebo	1	1	0	1.0	1	1	1	1.0
1	H	-	Yes	Placebo	1	1	0	1.0	4	1	1	1.0
1	A	-	Yes	Placebo	1	1	0	1.0	1	1	1	1.0
2a	J	+	N/A	450,000	1	828	827	827.8	9	10	1	1.1
2a	L	+	N/A	450,000	1	2855	2854	2854.7	9	32	22	3.4
2a	K	+	N/A	450,000	1	816	815	816.3	18	21	3	1.2
2b	R	+	Yes	900,000	1	5410	5409	5410.4	1	13	12	13.3
2b	Q	+	Yes	900,000	98	1450	1353	14.9	8	20	13	2.6
2b	O	+	Yes	900,000	1	1045	1044	1045.5	12	18	5	1.5
2b	*U*[2]	+	Yes	900,000	796	1412	616	1.8	1	7	6	7.0
2b	*N*[3]	+	Yes	900,000	1	600	599	600.0	5	15	11	3.3
2b	*S*[4]	+	N/A	900,000	1	1380	1379	1380.4	1	16	15	15.6
2b	P	+	Yes	Placebo	1	1	1	1.0	8	4	1	1.0
2b	T	+	Yes	Placebo	1	1	1	1.0	1	1	1	1.0
2b	M	+	Yes[5]	Placebo	238	103	1	1.0	4	4	1	1.1

[1]did not receive V5, no CHMI
[2]did not receive V3
[3]did not receive V5
[4]did not receive CHMI
[5]qPCR +, TBS -

Table S10: Increased levels as compared to pre-immunization for IgG and IgM antibodies to PfCSP by ELISA, IgG antibodies to PfSPZ by aIFA and inhibition of PfSPZ invasion of hepatocytes by aISI in sera taken 2 weeks after the 5th dose. For ELISA, samples were considered to have increased (seroconverted) over pre-immunization, if the difference between the post-immunization OD 1.0 and the pre-immunization OD 1.0 (net OD 1.0) was ≥50 and the ratio of the post-immunization OD 1.0 to pre-immunization OD 1.0 (ratio) was ≥3.0. For automated immunofluorescence assays, participants with a net reciprocal serum dilution for 2.0×10^5 arbitrary fluorescence units (AFU) of ≥150 and a ratio reciprocal serum dilution of ≥3.0 were considered positive. For the automated inhibition of sporozoite invasion assay, participants with a net reciprocal serum dilution for 80% inhibition of ≥10 in the inhibition of sporozoite invasion assay and a ratio reciprocal serum dilution for 80% inhibition of ≥3.0 in the inhibition of sporozoite invasion assay were considered positive.

	PfCSP IgG ELISA	PfCSP IgM ELISA	PfMSP5 IgG ELISA	PfSPZ aIFA	PfSPZ aISI
	2 weeks post 5th dose				
HIV- (no. with significant increase/no. vaccinated)	5/5 (100%)	5/5 (100%)	0/5 (0%)	5/5 (100%)	5/5 (100%)
HIV+ (no. with significant increase/no. vaccinated)	5/5 (100%)	5/5 (100%)	1/5 (20%)	4/5 (80%)	3/5 (60%)
HIV- and HIV+ Placebo (no. with significant increase/no. vaccinated)	0/3 (0%) (p=0.008)<br>0/3 (0%) (p=0.008)	1/3 (33%) (p=0.063)<br>0/3 (0%) (p=0.008)	0/3 (0%) (p>0.99)<br>0/3 (0%) (p>0.99)	0/3 (0%) (p=0.008)<br>0/3 (0%) (p=0.047)	0/3 (0%) (p=0.008)<br>0/3 (0%) (p=0.148)

Table S11: Inclusion Criteria

1. Male and female adults, from 18 to 45 years of age
2. Long term (at least two years) or permanent residence in the Bagamoyo district or nearby districts in Coastal and Dar-es-Salaam regions
3. Availability through mobile phone 24 hours a day during the whole study period
4. Ablility and willingness to complete the study visit schedule for safety follow-up and protocol compliance
5. Agreement to provide personal contact information and contact information of a third-party household member or close friend to study team
6. Agreement not to participate in any other clinical study involving investigational medicinal products during the study period, except enrollment in observational studies (such a co-enrollment must be approved by the PI)
7. Agreement to release medical and other information concerning contra-indications for participation in the study, and to be attended by a study clinician for physical examination and clinical investigations including electrocardiogram (ECG)
8. Willingness to undergo all blood, urine and stool tests (as specified in the protocol) and additional tests that may be ordered by the study clinician to rule-out significant abnormality(ies)
9. Female volunteers must be willing to take measures not to become pregnant if selected for participation in the trial and to undergo serum pregnancy test at screening and at defined time-points during the trial
10. Volunteers for enrollment into HIV positive sub-groups must have:
11. Documented HIV infection, be in general good health and on stable ART use for at least three (3) months, preferably six (6), prior to screening
12. WHO clinical stage 1 of HIV disease
13. CD4+ T-cell count >500 cells/μL at screening
14. Attending a care and treatment centre (CTC) within the study area for medical management of HIV infection, and agreeing to maintain regular attendance to such care and treatment centre while participating in the study
15. Agreement to allow the clinical team to contact and coordinate care with the volunteer's HIV CTC.
16. Correctly answering 10 out of 10 questions during informed consent process to demonstrate the understanding of study design, study procedures, risks and benefits
17. Signing and dating written informed consent, in accordance with local practice.

Table S12: Exclusion Criteria

1. Previous receipt of an investigational malaria vaccine or drug in the last 5 years
2. Receipt of standard vaccinations within 4 weeks prior to the first immunization with a PfSPZ product or are planning to take standard vaccinations during the trial through 4 weeks following the last injection with a PfSPZ product
3. Participation in any other clinical trial involving investigational medicinal products within 30 days prior to the onset of the study
4. Clinically significant cardiac abnormalities as indicated by history, physical examination or clinically significant abnormalities in electrocardiogram (ECG)
5. Positive family history in a 1st or 2nd degree relative for cardiac disease at age< 50 years old
6. A history of psychiatric disease
7. History of afebrile seizures, atypical febrile seizures or epilepsy
8. History of drug or alcohol abuse interfering with normal social function
9. History of chronic immunodeficiency condition (other than HIV) or autoimmune disease
10. The use of chronic immunosuppressive drugs, antibiotics, or other immune modifying drugs within three months prior to study onset (ART and inhaled and topical corticosteroids are allowed)
11. Meeting exclusion criteria based on the protocol algorithm for assessment of TB disease risk
12. Currently on Co-Trimoxazole (trimethoprim/sulfamethoxazole) prophylactic treatment (CPT)
13. Currently taking rifampin (isoniazid is not an exclusion criterion)
14. Body mass index (BMI) of <18 or >30 Kg/m2
15. Females who are pregnant (as indicated by postive serum pregnancy test), nursing, or plan on becoming pregnant or nurse within the duration of trial
16. Newly diagnosed with positive HIV infection at screening
17. Positive hepatitis (B or C virus) tests
18. Symptoms, physical signs and laboratory values suggestive of clinically significant systemic disorders or any other conditions which could interfere with the interpretation of the study results or compromise the health of the volunteers
19. Medical, social condition or occupational reason that, in the judgment of the investigator, is a contraindication to protocol participation, may impair the volunteer's ability to give informed consent or effectively participate in the study, may significantly increase the risk to the volunteer because of participation in the study or may impair interpretation of the study data

Table S13: Study schema/schedule of assessments:

Appendix (1A): Flow-chart Study Groups 1 &2b		Screening		Vaccination and Post-vaccination Follow-up																																	CHMI Phase	Unscheduled
Days Relative to First Subject First - DVI		(-120) to (-1)		0		+1	+2			+3	+4			+5	+6			+7	+8	+9	+10	+11	+12	+13	+19	+28			+29	+30	+31	+32	+33	+34	+35	+42	+49 to +105	
Visit Code		Sc1	Sc2	V❶			V❷				V❸				V❹											V❺											CHMI at +49	
				Pre	DVI	+24h	Pre	DVI	+2h	+1	Pre	DVI	+2h	+1	Pre	DVI	+2h	+1	+2	+3	+4	+5	+6	+7	+14	Pre	DVI	+2h	+1	+2	+3	+4	+5	+6	+7	+14	V5+21	
Window Period [Days or hours(h)]		—	—	-48h	—	±12h	-24h	—	—	—	-24h	—	—	—	-24h	—	—	—	±1	—	—	—	—	±2	±2	-48h	±7		—	±1	—	—	—	—	±2	±2	-7 to +70	
Visit Type	Group 1 [HIV -ve]	cL	cL	cL	W	W	cL	W	W	☎	cL	W	W	☎	cL	W	W	☎	cL	☎	☎	☎	☎	cL	cL	cL	W	W	☎	cL	☎	☎	☎	☎	cL	cL		[CL]
	Group 2b [HIV +ve]	cL	cL	cL	W	W	W	W	W	W	W	W	W	W	W	W	W	W	W	☎	☎	☎	☎	cL	cL	cL	W	W	☎	cL	☎	☎	☎	☎	cL	cL		
**Clinical Procedures**																																						
Informed Consent		✓																																				
Demographic Data		✓																																				
Medical History		✓																																				
Vital Signs		✓		✓		✓	✓		✓		✓		✓		✓		✓		✓					✓	✓	✓		✓		✓					✓	✓		
Height (at Sc1 Only) & Body Weight		✓																						✓											✓			
Review of TB risk Factors & HIV Clinical Staging*		✓																						✓											✓			
Drug History		✓		✓		✓	✓		✓	✓	✓		✓	✓	✓		✓	✓	✓	✓	✓	✓	✓	✓	✓	✓		✓	✓	✓	✓	✓	✓	✓	✓	✓	Please See Appendix 1C	[CL]
Birth Control Baseline and Follow-up [F]		✓		✓																				✓											✓			
Physical examination	• Detailed	✓																																				
	• ±Focused			✓		✓	✓		✓		✓		✓		✓		✓		✓					✓	✓	✓		✓		✓					✓	✓		
ECG		✓																																				
Review of Eligibility Criteria		✓	✓																																			
Adverse Events	Solicited [+Ruler & Thermometer Use]		DRT		S+L	S+L	S+L	S+L	S+L	S+L	S+L	S+L	S+L	S+L	S+L	S+L	S+L	S+L	S+L	S	S	S	S	S			S+L	S+L	S+L	S+L	S	S	S	S	S			
	Unsolicited				✓	✓	✓	✓	✓	✓	✓	✓	✓	✓	✓	✓	✓	✓	✓	✓	✓	✓	✓	✓	✓	✓	✓	✓	✓	✓	✓	✓	✓	✓	✓	✓		
	SAEs				✓	✓	✓	✓	✓	✓	✓	✓	✓	✓	✓	✓	✓	✓	✓	✓	✓	✓	✓	✓	✓	✓	✓	✓	✓	✓	✓	✓	✓	✓	✓	✓		
Insecticide Treated Net	Supply					✓																																
	Assessment on the use																							✓											✓			
**Laboratory Tests**		Numbers represent Blood Volume to Collect at each Time-Point																																				
Hematology (FBP) (0.5mL)		0.5		0.5			0.5				0.5				0.5				0.5					0.5		0.5				0.5					0.5			
Biochemistry (1mL)	ALT, AST, Bil.t, Creat, Glu, [T]	1																																				
	ALT, AST, Bil.t, Creat			1			1				1				1				1					1		1				1					1			
Parasitology	BS + PCR (1mL)⊕	1		1			1				1				1				1RT							1				1RT					1	1	Please See Appendix 1C	[CL]
Serology [HIV**, HBV and HCV] (1mL)		1																																				
Urine Dipstick [for Blood and Protein]		✓																																				
Stool and Urine for helminths***		✓																																				
Serum Sample Pregnancy Test (0.5 mL) [F]		0.5		0.5																						0.5												
Immunology	Humoral (5-10mL)			10											5									5	10	5										5		
	Cellular (50-100mL)			100																				50	50	50										50		
	Transcriptomics (2.5mL)			2.5			2.5				2.5				2.5				2.5					2.5	2.5	2.5				2.5					2.5			
HIV Related Tests (G1b & G2b)	CD4 T cell count (1 mL)	1																								1												
	Plasma HIV-1 RNA (2 mL)	2																								2												
	Cellular HIV-1 DNA (4 mL)	4																								4												
**Blood Volume per Scheduled Visit (mL)**		11		116			5				5				10				4					59	63	68				4					5	56		
Maximum Scheduled Blood Volume		***407 mL collected Over 10 weeks Period*** *[Screening, Vaccination and Post-vaccination Follow-up]* ***(Maximum allowed at any 8 week period is 550mL)***																																				

Legend Clinical Procedures

- V=Timepoint for DVI administration of PfSPZ vaccine ¦ W=Post DVI Ward Observation at BCTU ¦ cL=Clinic Visit at BCTU
- DRT=Practice Diary Cards, Ruler and Thermometer; to train and assess eligible volunteers knowledge on the use
- ☎=Telephone follow-up and Use of volunteers Diary Cards
- S=Systemic AE assessment with the use of Thermometer ¦ L=Local (at injection site) AEs assessment with the use of Rulers
- [CL]=On clinician request only and/ or as indicated in the protocol ¦ *=HIV Clinical Staging with review of CTC Documents (for G2 Volunteers only)

Legend - Laboratory Procedures

- [T]=Troponin sample will be collected but frozen for run in comparative assay if needed
- ⊕=Collect specimen for confirmed Malaria positive volunteers before treatment as follows: Parasite Differentiation Type assay (3mL)
- [F]=For female volunteers only ¦ RT=Prospective qPCR will be performed
- **=HIV serology will only be performed for volunteers whose HIV diagnosis is negative or unknown
- ***=May be collected within 2 days of visit

Appendix (1B): Flow-chart Study Group 2a

Appendix (1B): Flow-chart Study Group 2a	Screening		Vaccination and Post-vaccination Follow-up																																		Unscheduled
Days Relative to First Subject First - DVI	(-120) to (-1)		0		+1	+2			+3	+4			+5	+6			+7	+8	+9	+10	+11	+12	+13	+19	+28			+29	+30	+31	+32	+33	+34	+35	+42	+56	+84	
Visit Code	Sc1	Sc2	V❶			V❷				V❸				V❹											V❺													
			Pre	DVI	+24h	Pre	DVI	+2h	+1	Pre	DVI	+2h	+1	Pre	DVI	+2h	+1	+2	+3	+4	+5	+6	+7	+14	Pre	DVI	+2h	+1	+2	+3	+4	+5	+6	+7	+14	+28	+56	
Window Period [Days or hours(h)]	—	—	-48h	—	±12h	-24h	—	—	—	-24h	—	—	—	-24h	—	—	—	±1	—	—	—	—	±2	±2	-48h	±7		—	±1	—	—	—	—	±2	±2	±2	±2	[CL]
Visit Type	cL	cL	cL	W	W	W	W	W	W	W	W	W	W	W		W	W	W	☎	☎	☎	☎	cL	cL	cL	W	W	☎	cL	☎	☎	☎	☎	cL	cL	cL	cL	
**Clinical Procedures**																																						
Informed Consent	✓																																					
Demographic Data	✓																																					
Medical History	✓																																					
Vital Signs	✓		✓		✓	✓		✓		✓		✓		✓		✓		✓					✓	✓	✓		✓		✓					✓	✓	✓	✓	
Height (at Sc1 Only) & Body Weight	✓																						✓											✓		✓		
Review of TB risk Factors & HIV Clinical Staging*	✓																						✓											✓		✓	✓	
Drug History	✓		✓		✓	✓		✓	✓	✓		✓	✓	✓		✓	✓	✓	✓	✓	✓	✓	✓	✓	✓		✓	✓	✓	✓	✓	✓	✓	✓	✓	✓	✓	
Birth Control Baseline and Follow-up [F]	✓		✓																				✓											✓		✓		
Physical examination • Detailed	✓																																					
Physical examination • ±Focused			✓		✓	✓		✓		✓		✓		✓		✓		✓					✓	✓	✓		✓		✓					✓	✓	✓	✓	[CL]
ECG	✓																																					
Review of Eligibility Criteria	✓	✓																																				
Adverse Events: Solicited [+Ruler & Thermometer Use]		DRT		S+L	S+L	S+L	S+L	S+L	S+L	S+L	S+L	S+L	S+L	S+L	S+L	S+L	S+L	S+L	S	S	S	S	S			S+L	S+L	S+L	S+L	S	S	S	S	S		S		
Adverse Events: Unsolicited				✓	✓	✓	✓	✓	✓	✓	✓	✓	✓	✓	✓	✓	✓	✓	✓	✓	✓	✓	✓	✓	✓	✓	✓	✓	✓	✓	✓	✓	✓	✓	✓	✓	✓	
Adverse Events: SAEs				✓	✓	✓	✓	✓	✓	✓	✓	✓	✓	✓	✓	✓	✓	✓	✓	✓	✓	✓	✓	✓	✓	✓	✓	✓	✓	✓	✓	✓	✓	✓	✓	✓	✓	
Insecticide Treated Net: Supply					✓																																	
Insecticide Treated Net: Assessment on the use																							✓											✓		✓		
**Laboratory Tests**	Numbers represent Blood Volume to Collect at each Time-Point																																					
Hematology (FBP) (0.5mL)	0.5		0.5			0.5				0.5				0.5				0.5					0.5		0.5				0.5					0.5		0.5	0.5	
Biochemistry (1mL): ALT, AST, Bil.t, Creat, Glu, [T]	1																																					
Biochemistry (1mL): ALT, AST, Bil.t, Creat			1			1				1				1				1					1		1				1					1		1	1	
Parasitology: BS + PCR (1mL)⊕, RT	1		1			1				1				1									1		1									1	1	1	1	
Serology [HIV**, HBV and HCV] (1mL)	1																																					
Urine Dipstick [for Blood and Protein]	✓																																					
Stool and Urine for helminths***	✓																																					
Serum Sample Pregnancy Test (0.5 mL) [F]	0.5		0.5																						0.5													
Immunology: Humoral (5-10mL)			10											5									5	10	5										5		5	[CL]
Immunology: Cellular (50-100mL)			100																				50	50	50										50		50	
Immunology: Transcriptomics (2.5mL)			2.5			2.5				2.5				2.5				2.5					2.5	2.5	2.5				2.5					2.5		2.5		
HIV Related Tests (G1b & G2b): CD4 T cell count (1 mL)	1																								1											1	1	
HIV Related Tests (G1b & G2b): Plasma HIV-1 RNA (2 mL)	2																								2											2	2	
HIV Related Tests (G1b & G2b): Cellular HIV-1 DNA (4 mL)	4																								4											4	4	
**Blood Volume per Scheduled Visit (mL)**	11		116			5				5				10				4					60	63	68				4					5	56	12	65	
Maximum Scheduled Blood Volume	***482 mL collected Over 16 weeks Period*** [*Screening, Vaccination and Post-vaccination Follow-up*] ***(Maximum allowed at any 8 week period is 550mL)***																																					

Legend Clinical Procedures

V=Timepoint for DVI administration of PfSPZ vaccine ¦ **W**=Post DVI Ward Observation at BCTU ¦ **cL**=Clinic Visit at BCTU

DRT=Practice Diary Cards, Ruler and Thermometer; to train and assess eligible volunteers knowledge on the use

☎=Telephone follow-up and Use of volunteers Diary Cards

S=Systemic AE assessment with the use of **Thermometer** ¦ **L**=Local (at injection site) AEs assessment with the use of **Rulers**

[CL]=On clinician request only and/ or as indicated in the protocol ¦ *=HIV Clinical Staging with review of CTC Documents (for G2 Volunteers only)

Legend - Laboratory Procedures

[T]=Troponin sample will be collected but frozen for run in comparative assay if needed

⊕=Collect specimen for confirmed Malaria positive volunteers before treatment as follows: Parasite Differenti

[F]=For female volunteers only ¦ **RT**=Prospective qPCR will be performed

**=HIV serology will only be performed for volunteers whose HIV diagnosis is negative or unknown

***=May be collected within 2 days of visit

Appendix(1C): Flow-chart for CHMI	CHMI and Post-CHMI Follow-up																								
Days Relative to V1	+49 (-7 to +70)			+50	+51	+52	+53	+54	+55	+56	+57	+58	+59	+60	+61	+62	+63	+64	+65	+66	+67	+68	+69	+77	+105
		CHMI																							
Visit Code	Pre	CH	CH+2h	CH+1	CH+2	CH+3	CH+4	CH+5	CH+6	CH+7	CH+8	CH+9	CH+10	CH+11	CH+12	CH+13	CH+14	CH+15	CH+16	CH+17	CH+18	CH+19	CH+20	+28	+56
Window Period (Days or hours (h))	-48h		—	—	—	—	—	—	—	—	—	—	—	—	—	—	—	—	—	—	—	—	—	±2	±2
CHMI Monitoring	cL	DVI	W	☎	☎	☎	☎	☎	☎	☎	W	W	W	W	W	W	W	W	W	W	W	W	W	cL	cL
**Clinical Procedures**																									
Vital Signs	✓		✓								✓	✓x2	✓x2	✓x2	✓x2	✓x2	✓x2	✓	✓	✓	✓	✓	✓	✓	✓
Body Weight																									✓
Review of TB risk Factors & HIV Staging*	✓																							✓	✓
Drug History	✓		✓	✓	✓	✓	✓	✓	✓	✓	✓	✓	✓	✓	✓	✓	✓	✓	✓	✓	✓	✓	✓	✓	✓
Birth Control Follow-up ⊡	✓																								✓
±Focused Physical Examination	✓		✓								✓	✓	✓	✓	✓	✓	✓	✓	✓	✓	✓	✓	✓	✓	✓
**Adverse Events Assessment** – Solicited [+Ruler & Thermometer Use]		S+L	S+L	S+L	S+L	S	S	S	S	S	S	S	S	S	S	S	S	S	S	S	S	S	S	S	
**Adverse Events Assessment** – Unsolicited		✓	✓	✓	✓	✓	✓	✓	✓	✓	✓	✓	✓	✓	✓	✓	✓	✓	✓	✓	✓	✓	✓	✓	
**Adverse Events Assessment** – SAEs	✓	✓	✓	✓	✓	✓	✓	✓	✓	✓	✓	✓	✓	✓	✓	✓	✓	✓	✓	✓	✓	✓	✓	✓	✓
**Insecticide Treated Net** – Assessment on the use																									✓
**Laboratory Tests**	Numbers represent Blood Volume to Collect at each Time-Point																								
**Hematology** (FBP) (0.5mL)	0.5											0.5												0.5	0.5
**Biochemistry**¦ ALT, AST,Bil.tot,Crea (1mL)	1											1												1	1
**Parasitology**¦ BS + PCR (1mL)⊕	1RT										1	2	2	2	2	2	2	1	1	1	1	1	1	1	1
**Serum Sample Pregnancy Test** (0.5 mL) ⊡	0.5																								0.5
**Detection of Antimalarial Drugs** (5mL)											5														
**Immunology** – Humoral (5-10mL)	10																						10	10	10
**Immunology** – Cellular (50-100mL)	50										50						50⊕						50	50	50
**Immunology** – Transcriptomics (2.5mL)	2.5										2.5						2.5⊕						2.5	2.5	2.5
**HIV Related Tests (G1b & G2b)** – CD4 T cell count (1 mL)	1										1													1	1
**HIV Related Tests (G1b & G2b)** – Plasma HIV-1 RNA (2 mL)	2										2													2	2
**HIV Related Tests (G1b & G2b)** – Cellular HIV-1 DNA (4 mL)	4										4													4	4
Blood Volume per Scheduled Visit (mL)	72										66	4	2	2	2	2	2	1	1	1	1	1	64	72	73
Total Scheduled Blood Volume (mL)	***365 mL collected Over 8-week Period*** *[Pre-CHMI, CHMI and Post-CHMI Follow-up]* ***(Maximum allowed at any 8 week period is 550mL)***																								

Legend Clinical Procedures	Legend - Laboratory Procedures
**CH**=Timepoint for DVI administration of PfSPZ-CHMI ¦ **W**=Post DVI Ward Observation at BCTU ¦ **cL**=Clinic Visit at BCTU	⊕=Collect specimen for confirmed Malaria positive volunteers before treatment as follows: *Parasite Differentiation Type assay (3mL) and Immunological assays (volumes indicated)*
(☎=Telephone follow-up and and Use of volunteers **Diary Cards**	⊡=For female volunteers only ¦ **RT**=Prospective qPCR will be performed
**S**=Systemic AE assessment with the use of **Thermometer** ¦ **L**=Local (at injection site) AEs assessment with the use of **Rulers**	
*=HIV Staging with review of CTC Documents (for G1b and 2b Volunteers only)	

Table S14: Disclosures, conflict of interest and patents.

LWPC, NK, PR, YA, TM, ERJ, PFB, BKLS, TLR, and SLH are (or were) all salaried, full-time employees of Sanaria, the developer and sponsor of the Sanaria PfSPZ Vaccine. BKLS and SLH are owners of Sanaria. BKLS, ERJ, and SLH are inventors on the following patents and applications for patent, which have been assigned to Sanaria. Co-authors who are inventors aree shown in bold.

8. **Title:** Apparatuses and Methods for the Production of Haematophagous Organisms and Parasites Suitable for Vaccine Production
   Inventors: **Hoffman** and Luke
   Date of Filing: 04/Oct 2004. Date of Issue: 12/June 2007
   US Patent Issue Number: 7,229,627

9. **Title:** Apparatuses and Methods for the Production of Haematophagous Organisms and Parasites Suitable for Vaccine Production
   Inventors: **Hoffman** and Luke
   Date of Filing: 21/Mar 2007. Date of Issue: 12/Aug 2014
   US Patent Issue Number: 8,802,919

10. **Title:** Apparatuses and Methods for the Production of Haematophagous Organisms and Parasites Suitable for Vaccine Production
    Inventors: **Hoffman** and Luke
    Date of Filing: 07/April 2003. Date of Issue: 20/August 2008
    EP Patent Issue Number: 1492402 (VALIDATED IN: Switzerland, France, UK, Netherlands, Romania, Slovenia)

11. **Title:** Apparatuses and Methods for the Production of Haematophagous Organisms and Parasites Suitable for Vaccine Production
    Inventors: **Hoffman** and Luke
    Date of Filing: 07/April 2010. Date of Issue: 24/Sept 2014
    EP Patent Issue Number: 2000026 (VALIDATED IN: Switzerland, France, UK, Netherlands, Slovenia, Germany, Ireland, Sweden, Monaco, Luxembourg, Italy, Spain, Austria, Denmark, Belgium, Finland)

12. **Title:** Apparatuses and Methods for the Production of Haematophagous Organisms and Parasites Suitable for Vaccine Production
    Inventors: **Hoffman** and Luke
    Date of Filing: 07/April 2003. Date of Issue: 31/Jul 2014
    Israeli Patent Issue Number: 164384

15. **Title:** Attenuated Plasmodium Sporozoite Parasite, Pharmaceutical Compositions and Vaccines Comprising the Same and Uses Thereof for the Prevention of Malaria
    Inventors: **Hoffman** and Luke
    Date of Filing: 20/Nov 2003. Date of Issue: 29/July 2011
    Israeli Patent Issue Number: 168651

26. **Title**: Methods for the Prevention of Malaria
    Inventors: **Hoffman** and Luke
    Date of Filing: 20/Nov 2003. Date of Issue: 29/Jul 2011
    Israel Patent Issue Number: 168651

29. **Title:** Purified *Plasmodium* and Vaccine Compositions
Inventors: **Sim**, Li, Stafford, **Hoffman**
Date of Filing: 08/Jan 2010. Date of Issue: 25/Oct 2011
US Patent Issue Number: 8,043,625

30. **Title:** Purified *Plasmodium* and Vaccine Compositions
Inventors: **Sim**, Li, Stafford, **Hoffman**
Date of Filing: 27/Aug 2010. Date of Issue: 5/Feb 2013
US Patent Issue Number: 8,367,810

31. **Title:** Purified *Plasmodium* and Vaccine Compositions
Inventors: **Sim**, Li, Stafford, **Hoffman**
Date of Filing: 28/Dec 2012. Date of Issue: 2/Sep 2014
US Patent Issue Number: 8,821,896

32. **Title:** Purified *Plasmodium* and Vaccine Compositions
Inventors: **Sim**, Li, Stafford, **Hoffman**
Date of Filing: 28/Dec 2012. Date of Issue: 31/Mar 2015
US Patent Issue Number: 8,992,944

33. **Title:** Purified *Plasmodium* and Vaccine Compositions
Inventors: **Sim**, Li, Stafford, **Hoffman**
Date of Filing: 27/Mar 2015. Date of Issue: 26/Jan 2016
US Patent Issue Number: 9,241,962

34. **Title:** Purified *Plasmodium* and Vaccine Compositions
Inventors: **Sim**, Li, Stafford, **Hoffman**
Date of Filing: 21/Jan 2016. Date of Issue: 11/Apl 2017
US Patent Issue Number: 9,616,115

35. **Title:** Purified *Plasmodium* and Vaccine Compositions
Inventors: **Sim**, Li, Stafford, **Hoffman**
Date of Filing: 14/Mar 2017. Date of Issue: 30/Apl 2019
US Patent Issue Number: 10,272,146

36. **Title:** Purified *Plasmodium* and Vaccine Compositions
Inventors: **Sim**, Li, Stafford, **Hoffman**
Date of Filing: 08/Jan 2010. Date of Issue: 18/Mar 2015
ARIPO Patent Issue Number: AP3192

37. **Title:** Purified *Plasmodium* and Vaccine Compositions
Inventors: **Sim**, Li, Stafford, **Hoffman**
Date of Filing: 08/Jan 2010. Date of Issue: 08/Jan 2015
Australian Patent Issue Number: 2010204887

38. **Title:** Purified *Plasmodium* and Vaccine Compositions
Inventors: **Sim**, Li, Stafford, **Hoffman**
Date of Filing: 08/Jan 2010. Date of Issue: 16/Aug 2017

European Patent Issue Number: 2387416 (VALIDATED IN: Austria, Belgium, Denmark Finland, France, Germany, Ireland, Italy, Luembourg, Monaco, Netherlands, Norway, Portugal, Romania, Slovenia, Spain, Sweden, Switzerland, United Kingdom)

39. **Title:** Purified *Plasmodium* and Vaccine Compositions
Inventors: **Sim**, Li, Stafford, **Hoffman**
Date of Filing: 04/May 2012. Date of Issue: 19/Dec 2014
Hong Kong Patent Issue Number: HK1163546

40. **Title:** Purified *Plasmodium* and Vaccine Compositions
Inventors: **Sim**, Li, Stafford, **Hoffman**
Date of Filing: 08/Jan 2010. Date of Issue: 29/Sep 2018
Israel Patent Issue Number: 213929

41. **Title:** Purified *Plasmodium* and Vaccine Compositions
Inventors: **Sim,** Li, Stafford, **Hoffman**
Date of Filing: 08/Jan 2010. Date of Issue: 01/May 2019
India Patent Issue Number: 312113

42. **Title:** Purified *Plasmodium* and Vaccine Compositions
Inventors: **Sim**, Li, Stafford, **Hoffman**
Date of Filing: 08/Jan 2010. Date of Issue: 01/May 2015
Japan Patent Issue Number: 5738199

43. **Title:** Purified *Plasmodium* and Vaccine Compositions
Inventors: **Sim**, Li, Stafford, **Hoffman**
Date of Filing: 08/Jan 2010. Date of Issue: 27/Jul 2017
South Korea Patent Issue Number: 101764142

44. **Title:** Purified *Plasmodium* and Vaccine Compositions
Inventors: **Sim**, Li, Stafford, **Hoffman**
Date of Filing: 08/Jan 2010. Date of Issue: 20/Jul 2017
Singapore Patent Issue Number: 2014400306-5

45. **Title:** Purified *Plasmodium* and Vaccine Compositions
Inventors: **Sim**, Li, Stafford, **Hoffman**
Date of Filing: 14/Jan 2010. Date of Issue: 21/Jan 2016
TaiwanPatent Issue Number: I-517858

46. **Title:** Purified *Plasmodium* and Vaccine Compositions
Inventors: **Sim**, Li, Stafford, **Hoffman**
Date of Filing: 08/Jan 2010. Date of Issue: 12/Sept 2011
Nigerian Patent Issue Number: RP NG/C/2011/429

47. **Title:** Purified *Plasmodium* and Vaccine Compositions
Inventors: **Sim**, Li, Stafford, **Hoffman**
Date of Filing: 08/Jan 2010. Date of Issue: 08/Jan 2014
Chinese Patent Issue Number: ZL201080004734.7

48. **Title:** Purified *Plasmodium* and Vaccine Compositions

Inventors: **Sim**, Li, Stafford, **Hoffman**
Date of Filing: 08/Jan 2010. Date of Issue: 04/Jul 2014
Indonesian Patent Issue Number: IDP000036235

49. **Title:** Purified *Plasmodium* and Vaccine Compositions
Inventors: **Sim**, Li, Stafford, **Hoffman**
Date of Filing: 08/Jan 2010. Date of Issue: 31/Oct 2012
South African Patent Issue Number: 2011/04897

50. **Title:** Purified *Plasmodium* and Vaccine Compositions
Inventors: **Sim**, Li, Stafford, **Hoffman**
Date of Filing: 08/Jan 2010. Date of Issue: 22/Sep 2020
Canadian Patent Issue Number: 2,749,262

51. **Title:** Pharmaceutical Compositions Comprising Attenuated Sporozoites and Glycolipid Adjuvants
Inventors: Chakravarty, **Hoffman,** Tsuji
Date of Filing: 28/ Oct 2013. Date of Issue: 08/Mar 2016
US Patent Issue Number: 9,278,125

52. **Title:** Pharmaceutical Compositions Comprising Attenuated Sporozoites and Glycolipid Adjuvants
Inventors: Chakravarty, **Hoffman,** Tsuji
Date of Filing: 18/Feb2016. Date of Issue: 09/May 2017
US Patent Issue Number: 9,642,909

53. **Title:** Infectious *Plasmodium* Sporozoites Grown In Vitro
Inventors: Eappen, **Hoffman**
Date of Filling: 11/Apl 2016. Date of Issue: 30/Jan 2018
US Patent Issue Number: 9,878,026

54. **Title:** Infectious *Plasmodium* Sporozoites Grown In Vitro
Inventors: Eappen, **Hoffman**
Date of Filling: 29/Dec 2017. Date of Issue: 15/Oct 2019
US Patent Issue Number: 10,441,646

55. **Title:** Infectious *Plasmodium* Sporozoites Grown In Vitro
Inventors: Eappen, **Hoffman**
Date of Filling: 05/Sep 2019. Date of Issue: 28/Dec 2021
US Patent Issue Number: 11,207,395

56. **Title:** Infectious *Plasmodium* Sporozoites Grown In Vitro
Inventors: Eappen, **Hoffman**
Date of Filling: 01/May 2015. Date of Issue: 18/Apl 2018
Nigeria Patent Issue Number: F/P/2016/394

57. **Title:** Infectious *Plasmodium* Sporozoites Grown In Vitro
Inventors: Eappen, **Hoffman**
Date of Filling: 01/May 2015. Date of Issue: 31/Jan 2018
OAPI Patent Issue Number: 18109

58. **Title:** Infectious *Plasmodium* Sporozoites Grown In Vitro
Inventors: Eappen, **Hoffman**
Date of Filling: 01/May 2015. Date of Issue: 15/Dec 2017
Hong Kong Patent Issue Number: 15786133.7

59. **Title:** Infectious *Plasmodium* Sporozoites Grown In Vitro
Inventors: Eappen, **Hoffman**
Date of Filling: 01/May 2015. Date of Issue: 01/May 2018
South African Patent Issue Number: 2016/07470

60. **Title:** Infectious *Plasmodium* Sporozoites Grown In Vitro
Inventors: Eappen, **Hoffman**
Date of Filling: 01/May 2015. Date of Issue: 15/Aprl 2020
ARIPO Patent Issue Number: AP5105

61. **Title:** Infectious *Plasmodium* Sporozoites Grown In Vitro
Inventors: Eappen, **Hoffman**
Date of Filling: 01/May 2015. Date of Issue: 04/Dec 2020
Australian Patent Issue Number: 2015252843

62. **Title:** Serum Antibody Assay for Determining Protection from Malaria
Inventors: Felgner, **Hoffman**, Seder, Campo
Date of Filing: 29/Dec 2017. Date of Issue: 05/Feb 2019
US Patent Issue Number: 10,197,577

65. **Title:** Mosquito Salivary Gland Extraction Device and Methods
Inventors: Taylor, Canezin, Schrum, Iordachita, Chirikjian, Laskowski, Chakravarty, **Hoffman**
Date of Filing: 13/Jun 2017. Date of Issue: 22/Sep 2020
US Patent Issue Number: 10,781,419

Figure S1: **Changes in CD4+ lymphocyte counts during immunization and CHMI in HIV+ subjects. Panel A**: three HIV infected participants in the pilot safety group received sequential doses of 4.5x10^5 PfSPZ on days 1, 3, 5, 7 and 29 (indicated by the green vertical arrows on the graph). No changes were noted in the CD4+ lymphocyte counts 3 weeks after the priming series of immunizations or 29 days after the booster. **Panel B**: nine additional HIV infected participants received sequential doses of 9.0x10^5 PfSPZ Vaccine (solid lines) or NS (dashed lines) on days 1, 3, 5, 7 and 29 (indicated by the green vertical arrows on the graph), followed by CHMI on day 51 (vertical red arrow). There was no indication that CD4+ lymphocyte counts declined in response to immunization. One participant ("T", a NS control) developed a late decline in CD4 count on day 106. This participant was PCR+ and TBS+ for *P. falciparum*, thought to represent a a new episode of parasitemia after treatment. This was associated with significant lymphopenia but with no change in the relative percentage of lymphocytes that were CD4+ (**panel C**), implying that this was not a CD4+ lymphocyte specific decline. The total lymphocyte and CD4+ lymphocyte counts rebounded rapidly after a repeat course of AL. HIV viral load remained below the limit of quantitation (40 copies/mL) at all time points.

A:

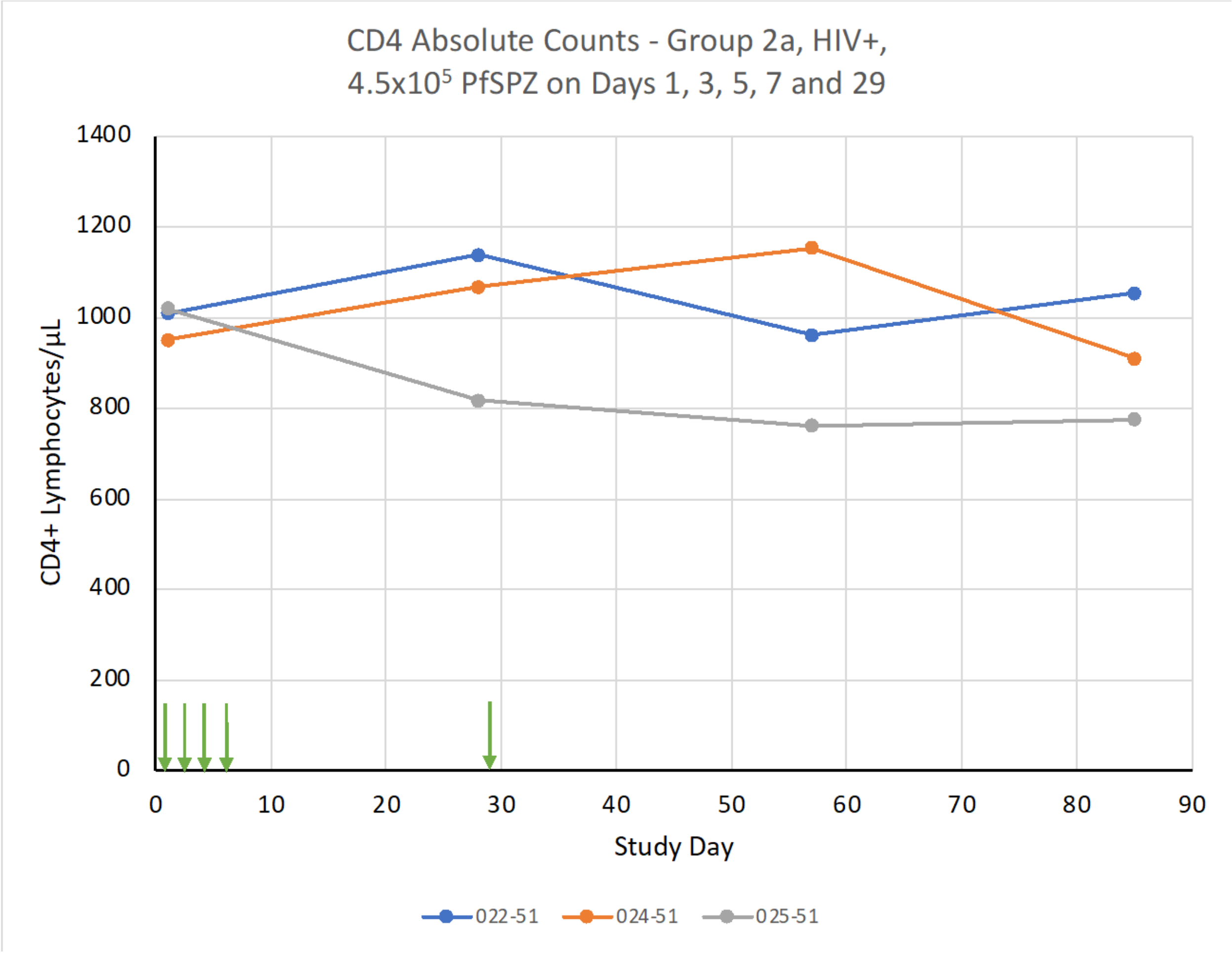

B.

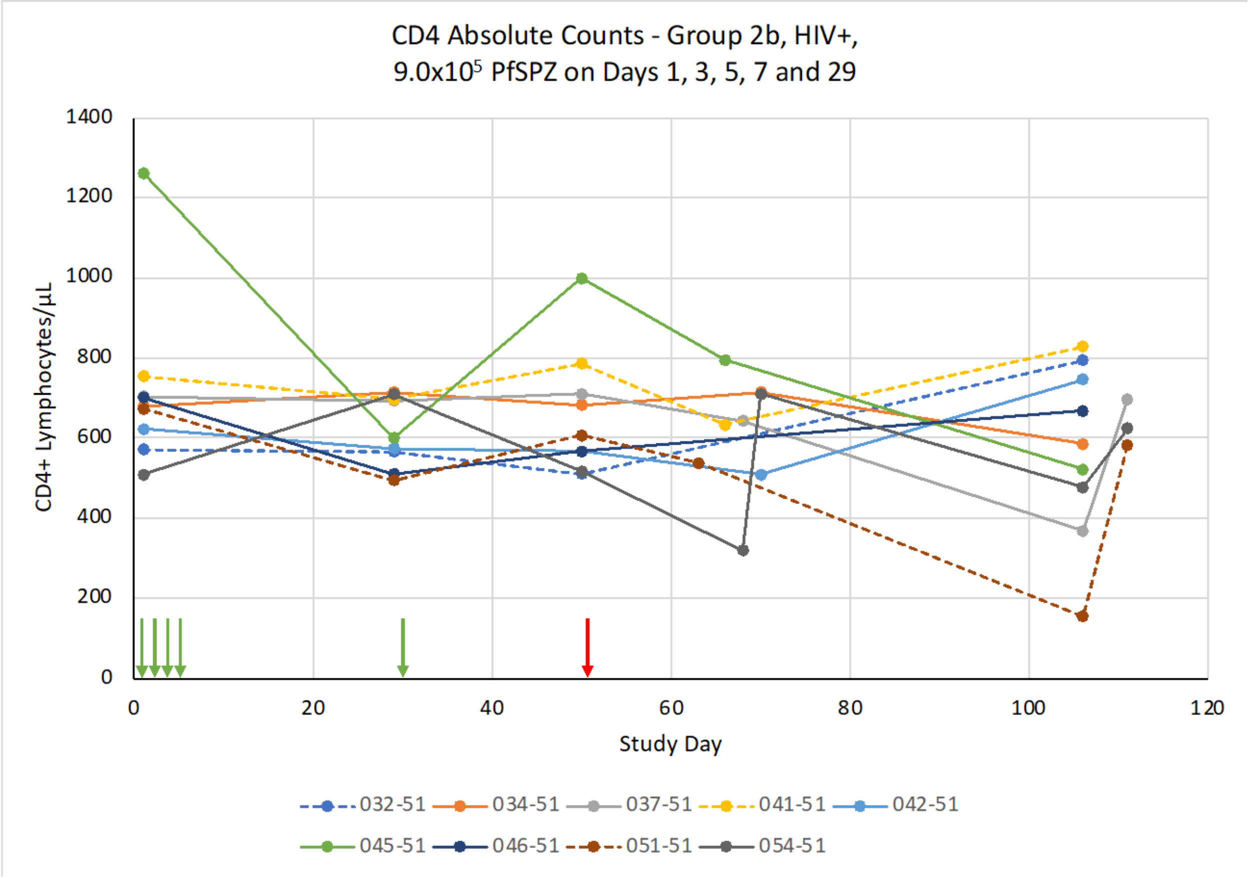

CD4 Absolute Counts - Group 2b, HIV+,
9.0x10^5 PfSPZ on Days 1, 3, 5, 7 and 29
CD4+ Lymphocytes/µL
1400
1200
1000
800
600
400
200
0
0
20
40
60
80
100
120
Study Day
032-51
034-51
037-51
041-51
042-51
045-51
046-51
051-51
054-51

C.

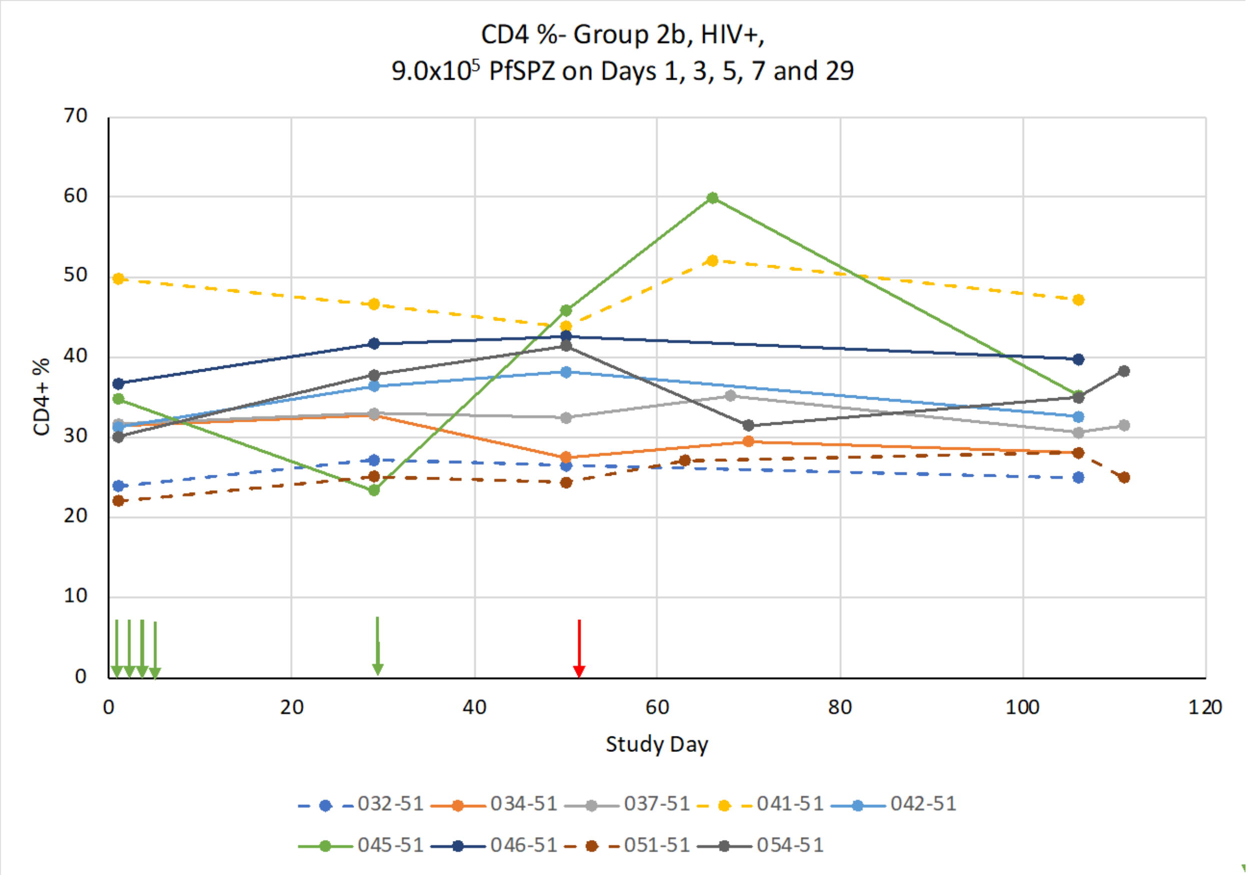

CD4 %- Group 2b, HIV+,
9.0x10^5 PfSPZ on Days 1, 3, 5, 7 and 29
70
60
50
40
30
20
10
0
CD4+ %
0
20
40
60
80
100
120
Study Day
032-51
034-51
037-51
041-51
042-51
045-51
046-51
051-51
054-51

Figure S2. Antibodies to PfMSP5. Antibodies assessed in sera taken 2 weeks after the final dose of PfSPZ Vaccine in participants who were uninfected (protected) (filled circles) and infected (open circles) during homologous CHMI with PfSPZ Challenge (NF54) administered 3 weeks after the final dose. Median and interquartile range of net OD 1.0 for IgG antibodies to PfMSP5 by ELISA. There was no significant difference between HIV- and HIV+ vaccinees for antibodies PfMSP5 by ELISA (Wilcoxon-Mann-Whitney test).

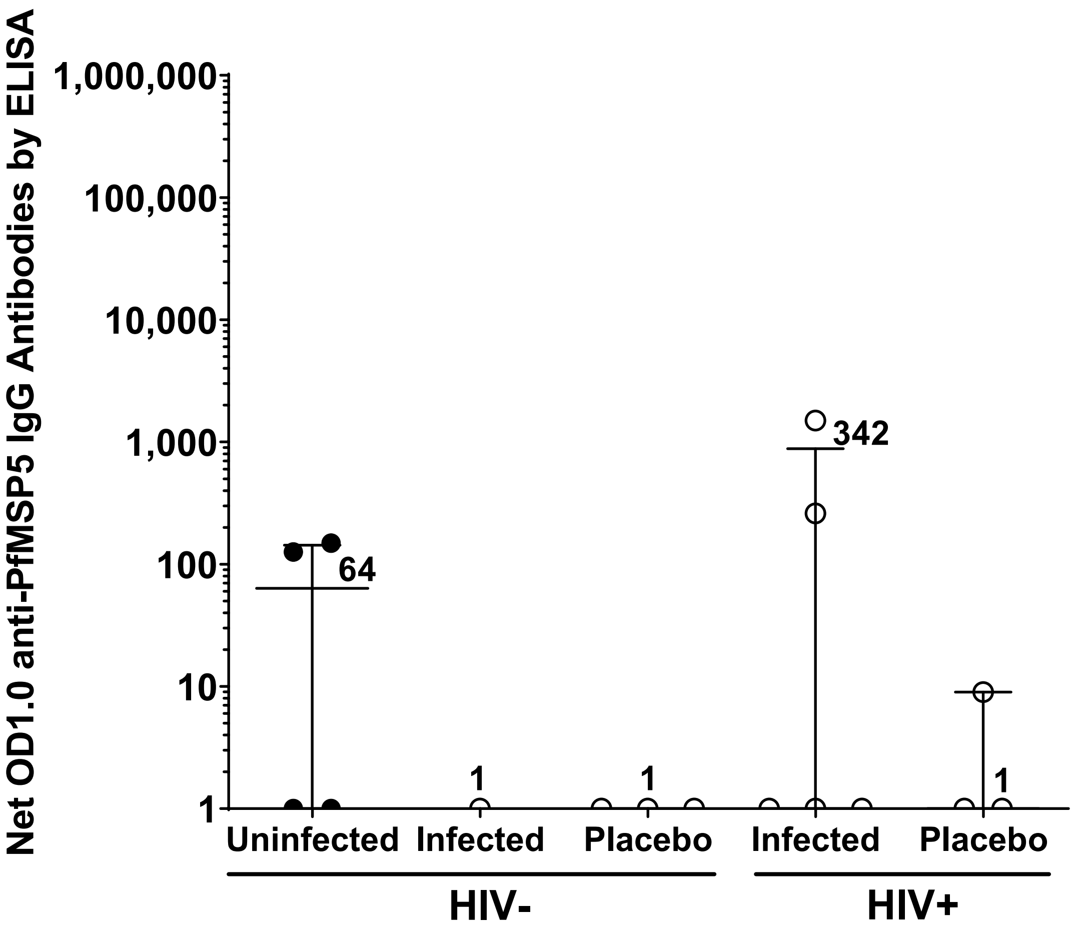

Figure S3. Antibodies to PfCSP and PfSPZ and functional activity of sera against PfSPZ. a) Median and interquartile range of ratio OD 1.0 for IgG and IgM antibodies to PfCSP by ELISA measured 2 weeks post 5th dose of PfSPZ Vaccine in HIV+ and HIV- Tanzanian adults who were uninfected (protected) and infected during CHMI administered 3 weeks after the 5th dose. b) Median and interquartile range of ratio IgG antibodies to PfSPZ by aIFA and net inhibition of PfSPZ invasion of hepatocytes by aISI measured 2 weeks post 5th dose of PfSPZ Vaccine in HIV+ and HIV- Tanzanian adults who were uninfected (protected) and infected during CHMI administered 3 weeks after the 5th dose. P values were calculated by Wilcoxon-Mann-Whitney test. For each panel, filled circles are uninfected subjects and open circles are infected subjects who received who received homologous CHMI with PfSPZ Challenge (NF54).

A

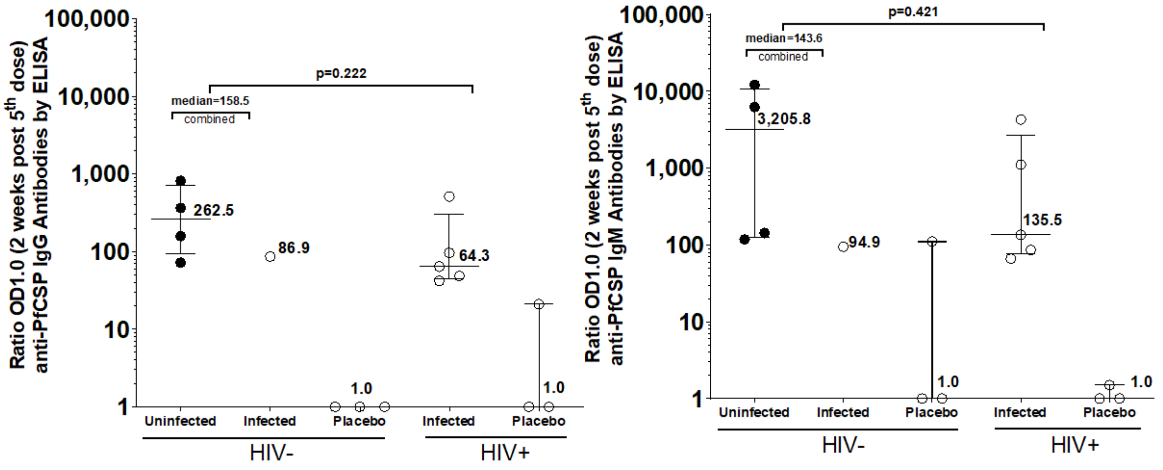

B

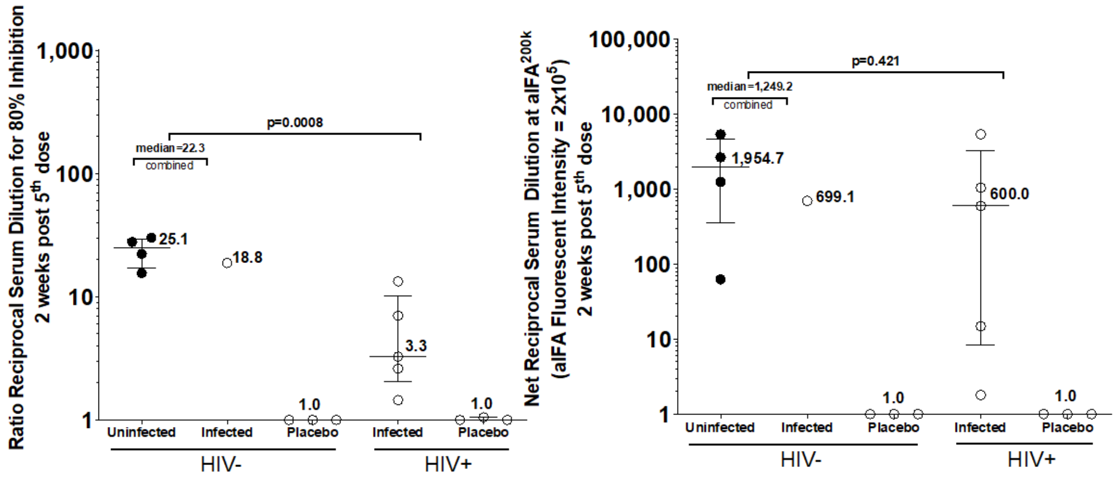

Figure S4: Box plots of all measured antibody features in Pre-Vaccination (Blue) and Post-Vaccination (Orange) samples for HIV negative and HIV positive vaccinees. Each row represents a separate Antigen and each column represents a different antibody detector including IgG Total, IgG1, IgG2, IgG3, IgM, IgA, Fcγ2A, Fcγ2B, Fcγ3A, Fcγ3B, FcAR, C1q, ADCD, ADCP, and ADNP. Mann Whitney U method was used for statistical testing of Post-Vaccination HIV Positive and HIV Negative groups. Statistical significance was represented as follows: * $p < 0.1$, p < ** 0.05, *** $p< 0.01$.

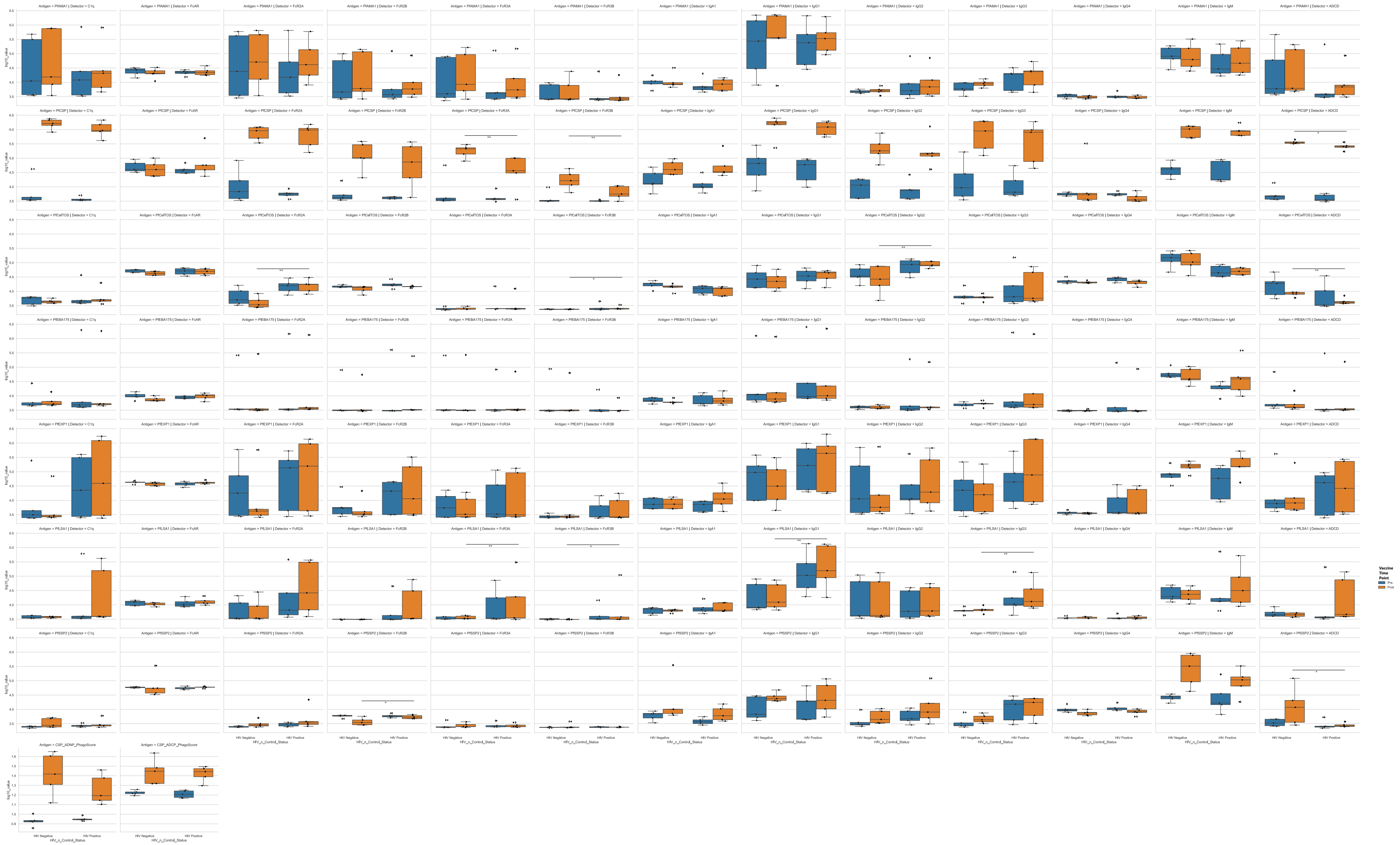

Figure S5: PfSPZ-specific memory CD8+ T cell responses pre and post immunization. Percent of memory CD8+ T cells in the blood expressing IFNγ, IL-2, or TNFα at pre-immunization or 2 weeks after the 4th and 5th doses of PfSPZ Vaccine ($9.0x10^5$) in HIV- (**panel A**) and HIV+ (**panel B**) participants. Results are the percentage of cytokine-producing cells after incubation with PfSPZ minus the percentage of cytokine-producing cells after incubation with vaccine diluent (medium with 1% human serum albumin). Bars indicate median values within each group and ● the individual participant data. One of 6 HIV negative participants demonstrated a positive response (defined as > 0.1% of CD8+ T cells expressing IFNγ, IL-2, or TNFα) two weeks after the 4th immunization (panel A). Two of 6 HIV+ participants demonstrated a positive response, one 2 weeks after the 4th immunization and a second 2 weeks after the 5th immunization.

A.

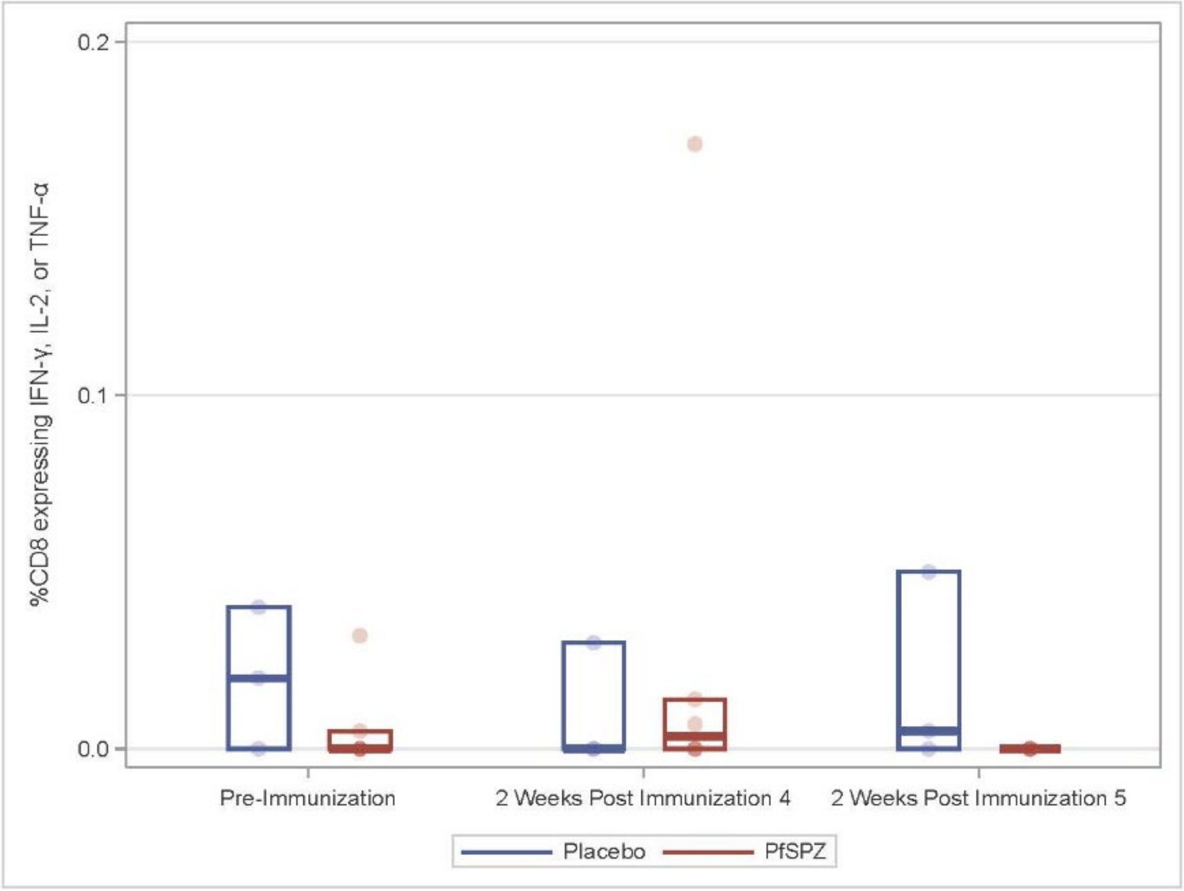

B.

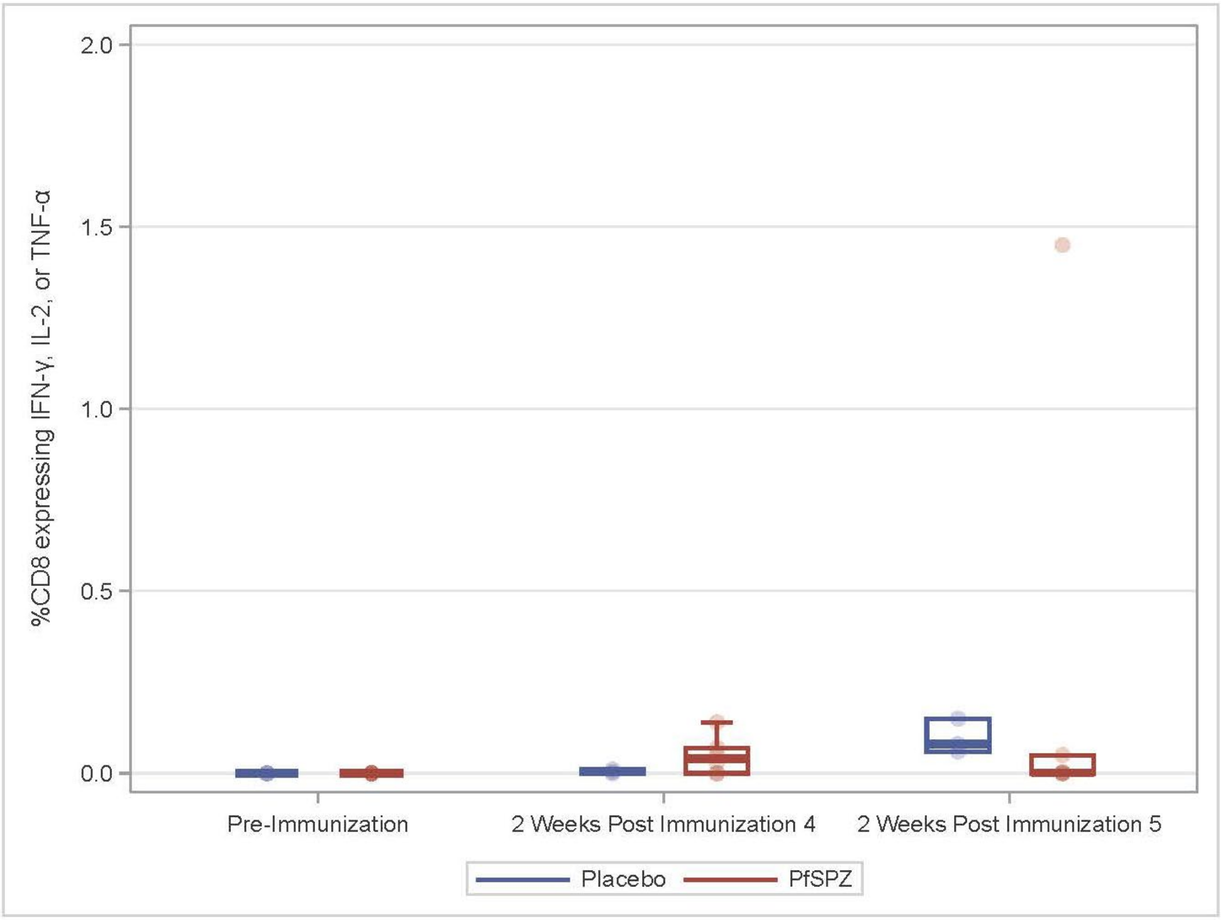
%CD8 expressing IFN-γ, IL-2, or TNF-α
2.0
1.5
1.0
0.5
0.0
Pre-Immunization
2 Weeks Post Immunization 4
2 Weeks Post Immunization 5
Placebo
PfSPZ

Figure S6: Individual participant CD4 (left panel) and CD8 (right panel) T cell responses to immunization. For each participant the percentage of memory CD4 cells positive for expression of IFN-γ, IL-2, TNF or CD154 was measured from samples obtained pre-immunization, 2 weeks after the initial 4 dose series of immunizations and 2 weeks after the 5th immunization after stimulation with PfSPZ Vaccine. For each participant the percentage of memory CD8 cells positive for expression of IFN-γ, IL-2, or TNF was measured from samples obtained pre-immunization, 2 weeks after the initial 4 dose series of immunizations and 2 weeks after the 5th immunization after stimulation with PfSPZ Vaccine. Responses to CMV were used a positive control.

Participant B (HIV-, PfSPZ Vaccine)

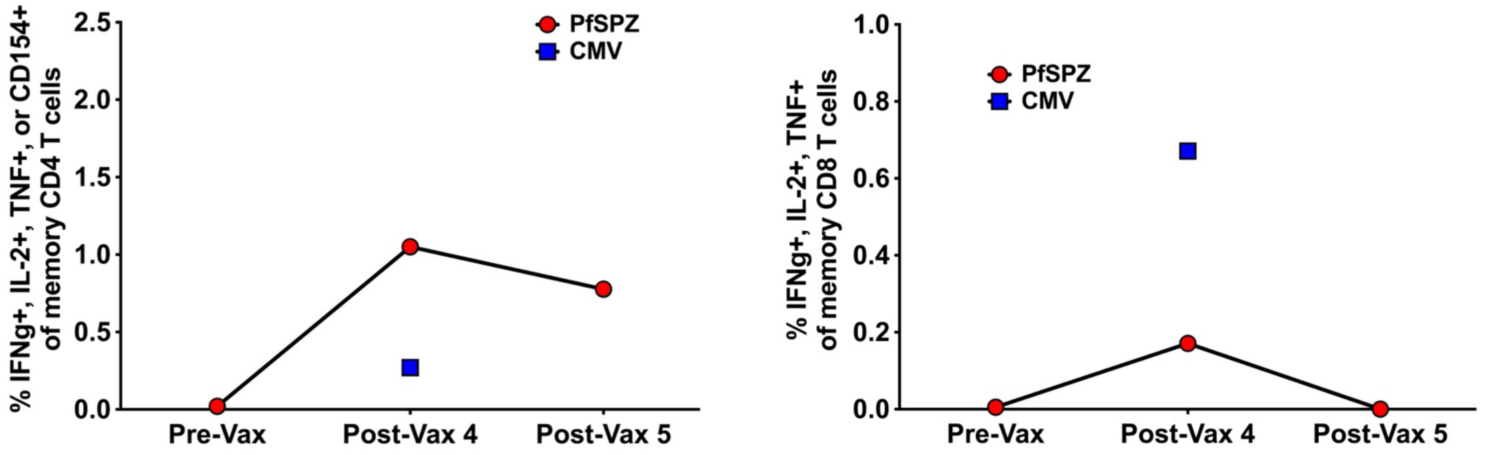

Participant C (HIV-, PfSPZ Vaccine)

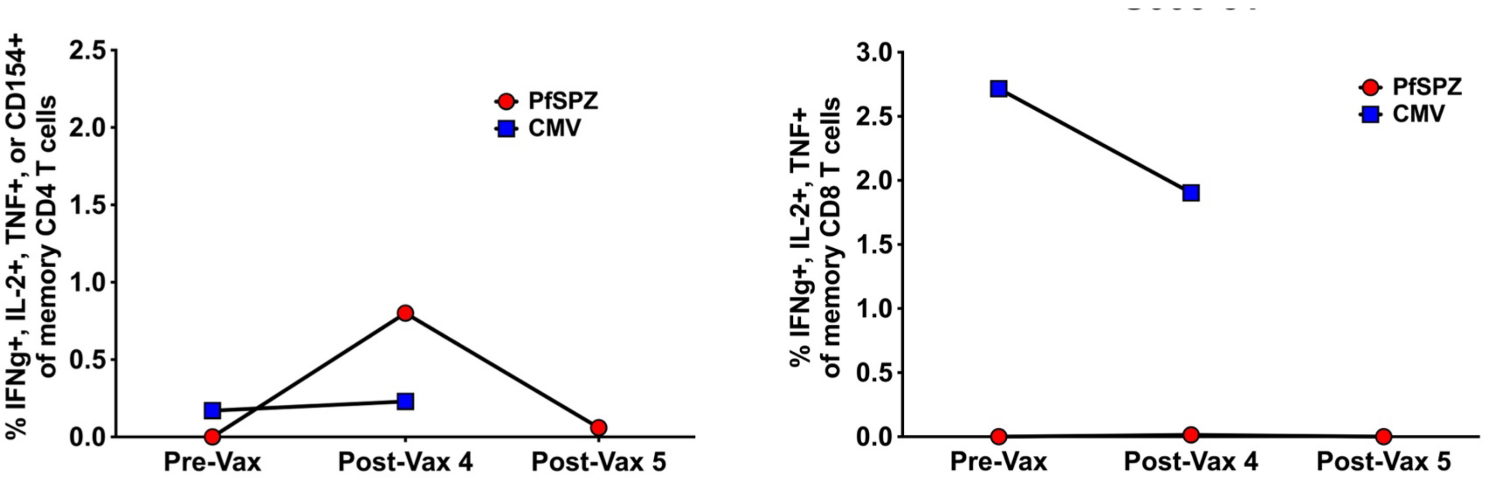

Participant D (HIV-, PfSPZ Vaccine)

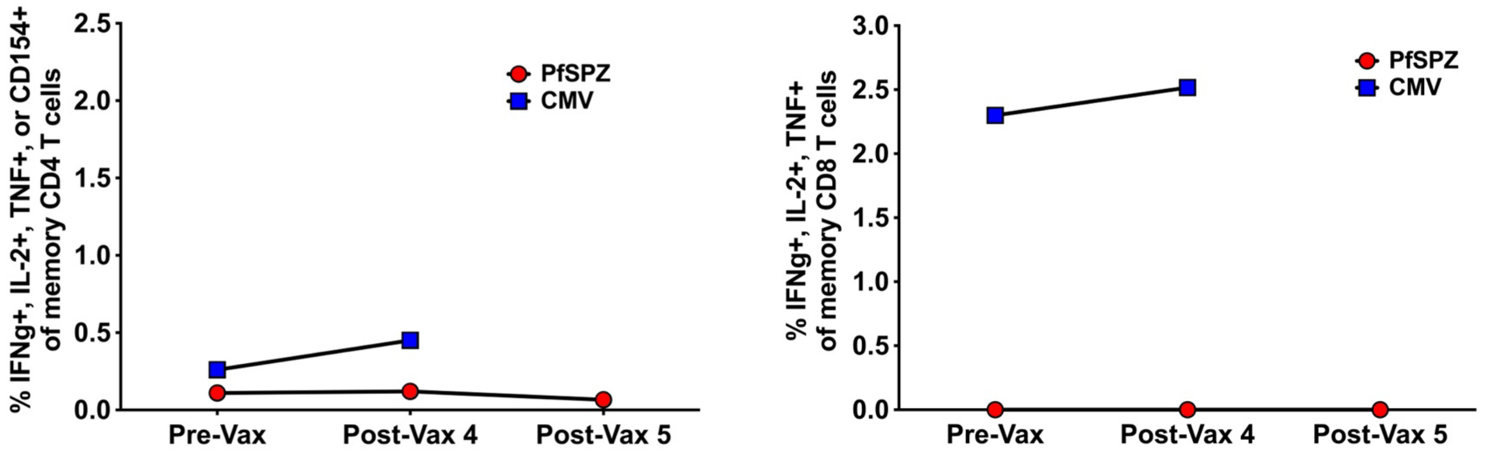

Participant F (HIV-, PfSPZ Vaccine)

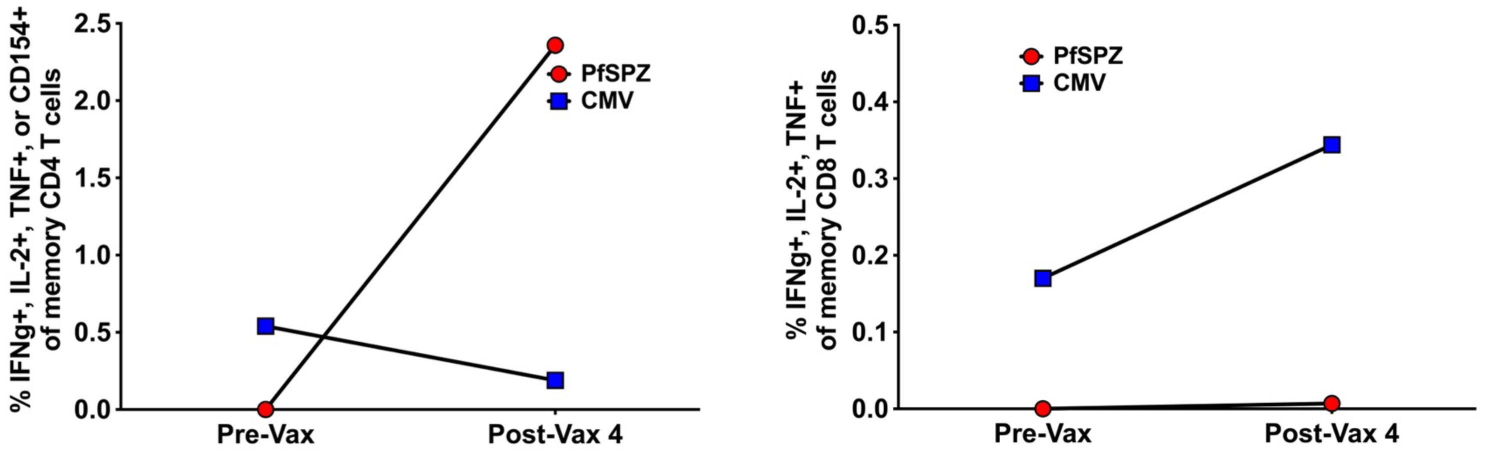

Participant G (HIV-, PfSPZ Vaccine)

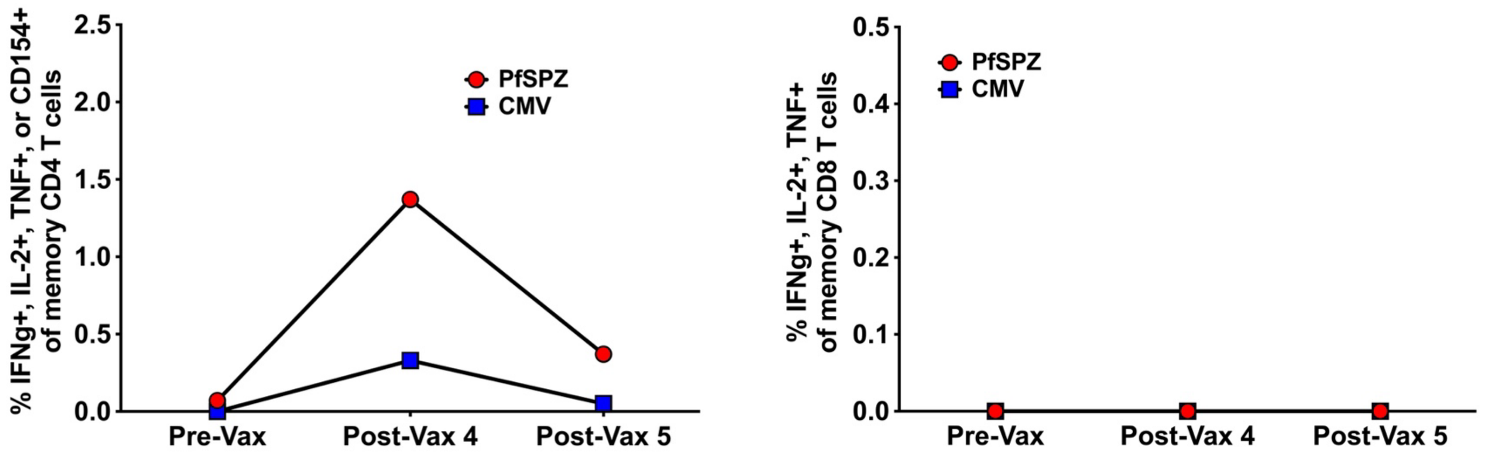

Participant I (HIV-, PfSPZ Vaccine)

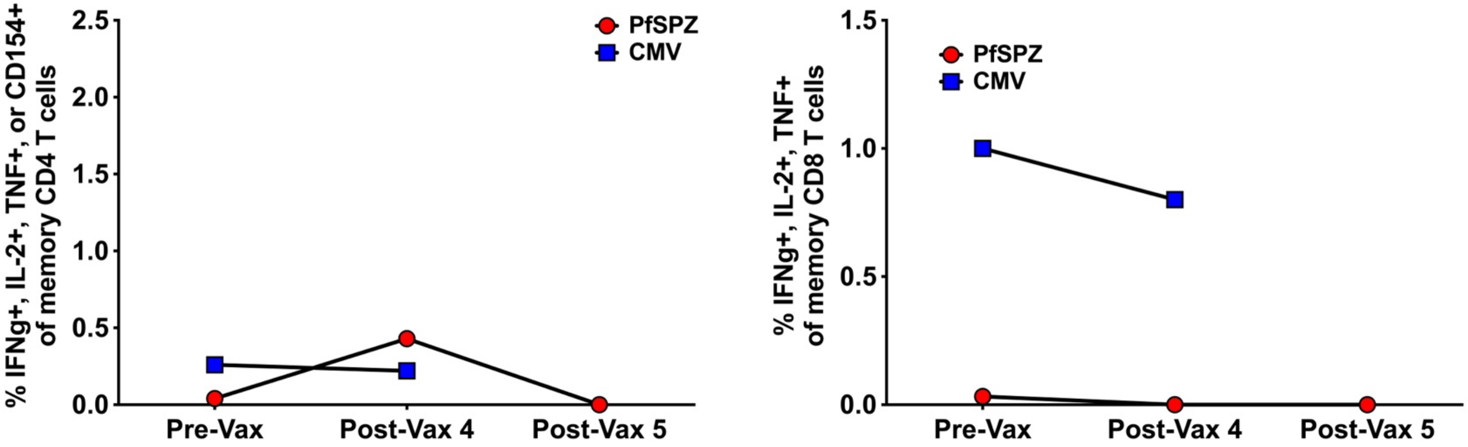

Participant A (HIV-, normal saline)

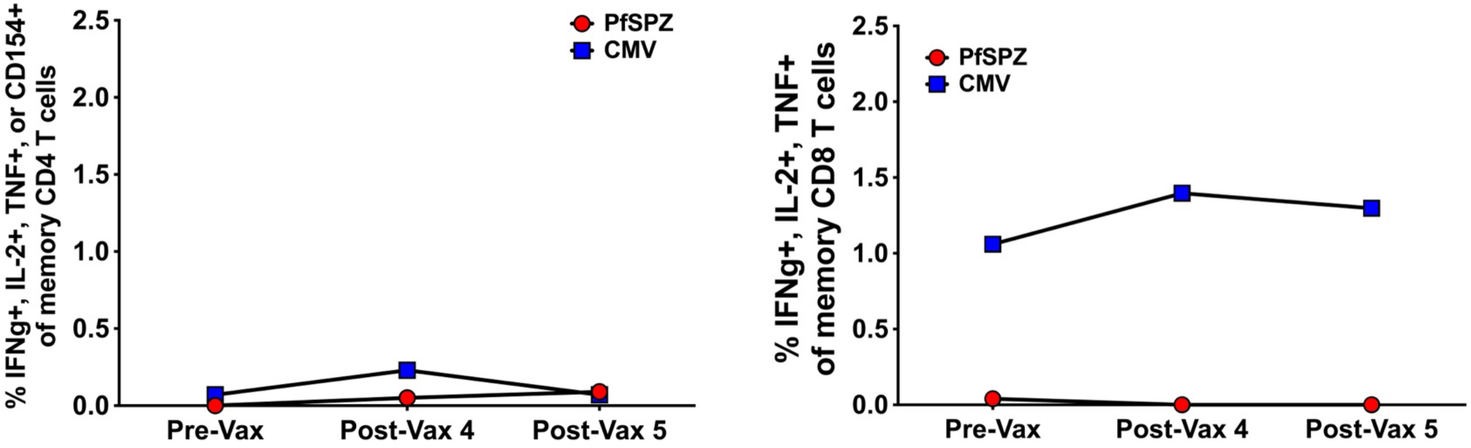

Participant E (HIV-, normal saline)

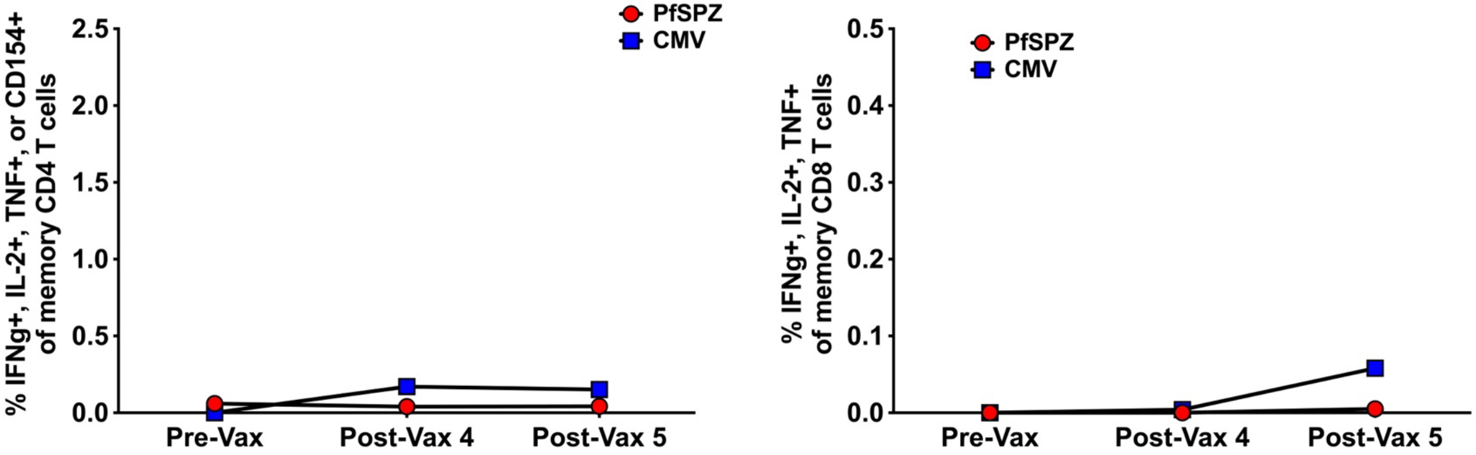

Participant H (HIV-, normal saline)

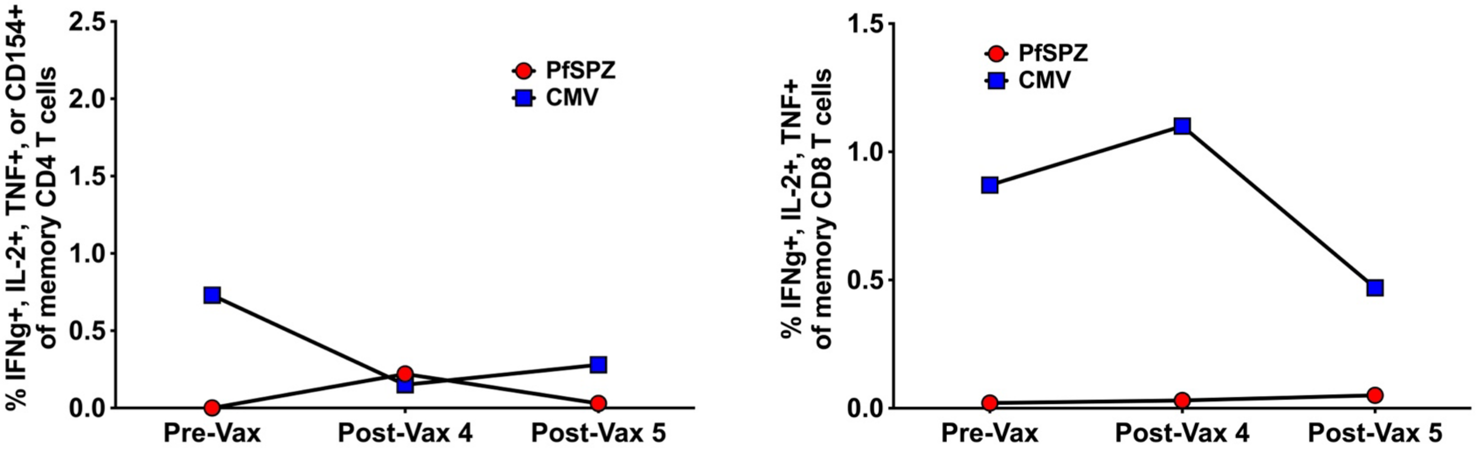

Participant N (HIV+, PfSPZ Vaccine)

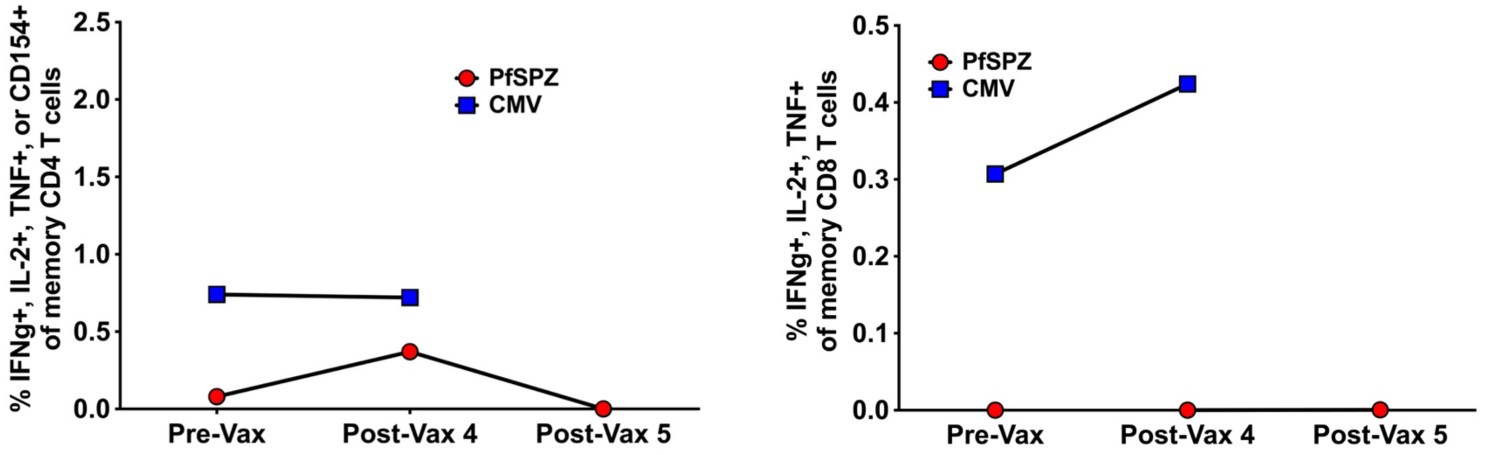

Participant O (HIV+, PfSPZ Vaccine)

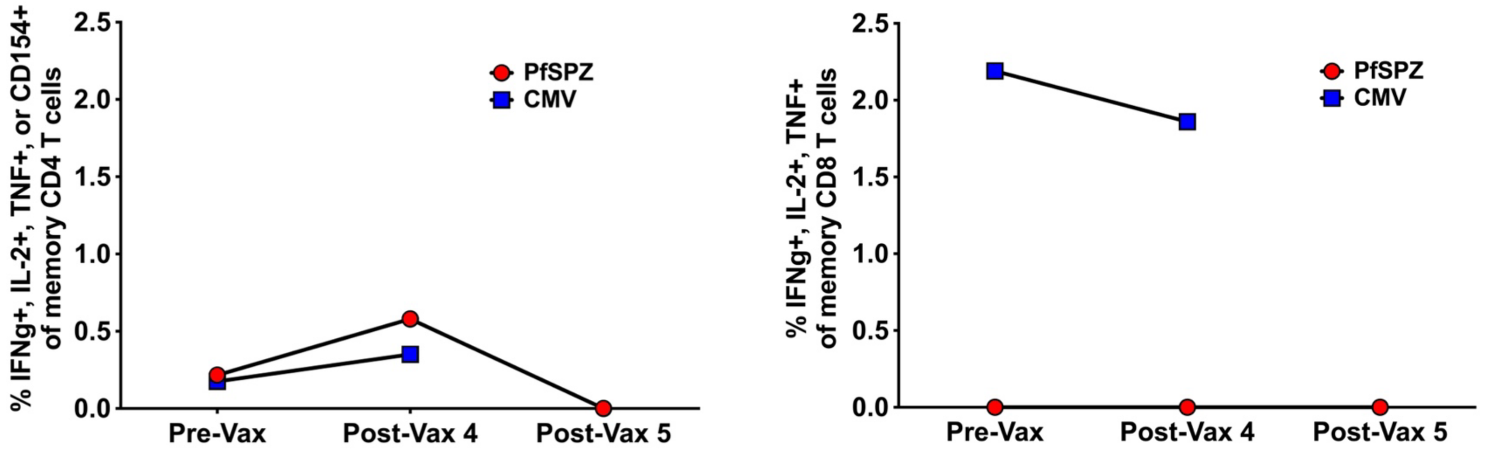

Participant Q (HIV+, PfSPZ Vaccine)

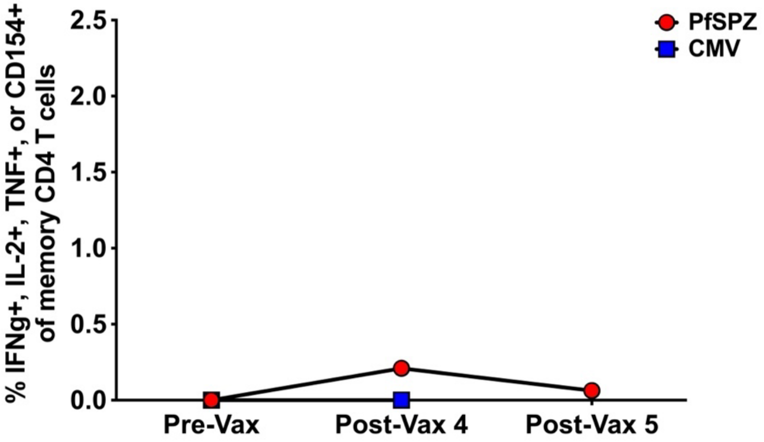

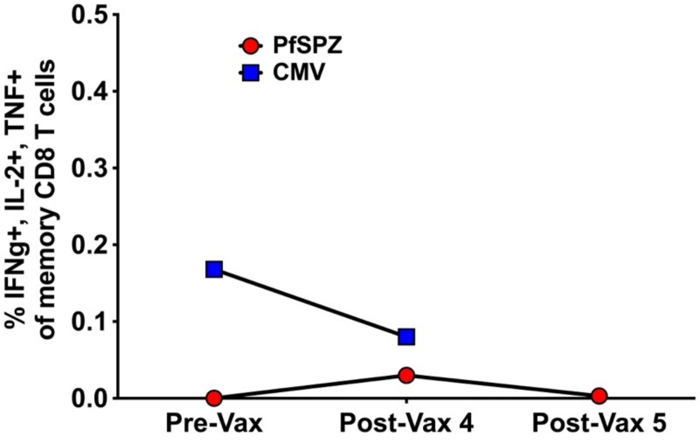

Participant R (HIV+, PfSPZ Vaccine)

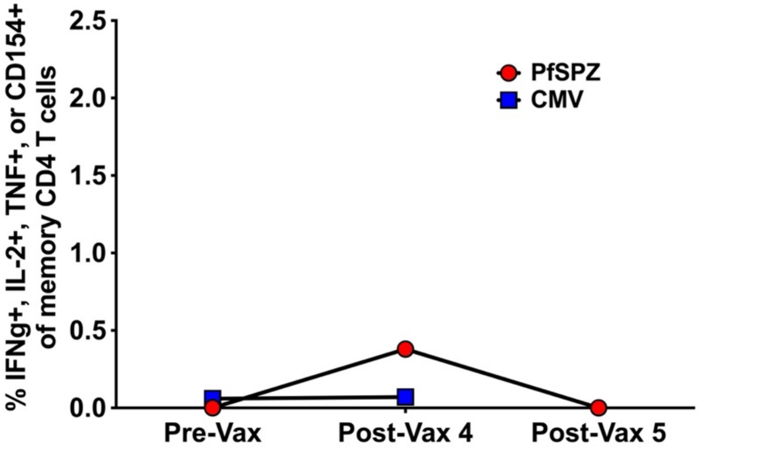

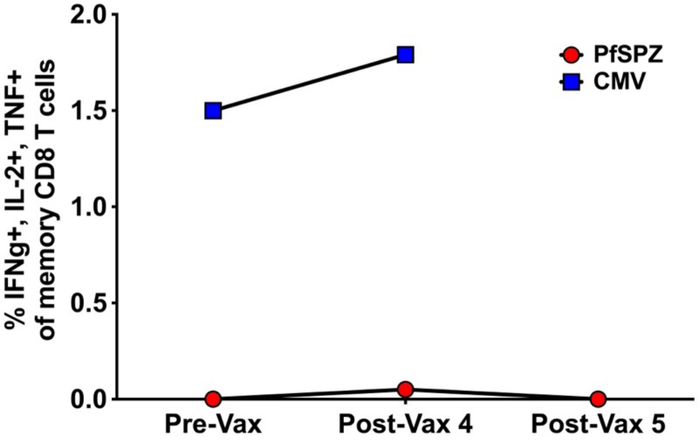

Participant S (HIV+, PfSPZ Vaccine)

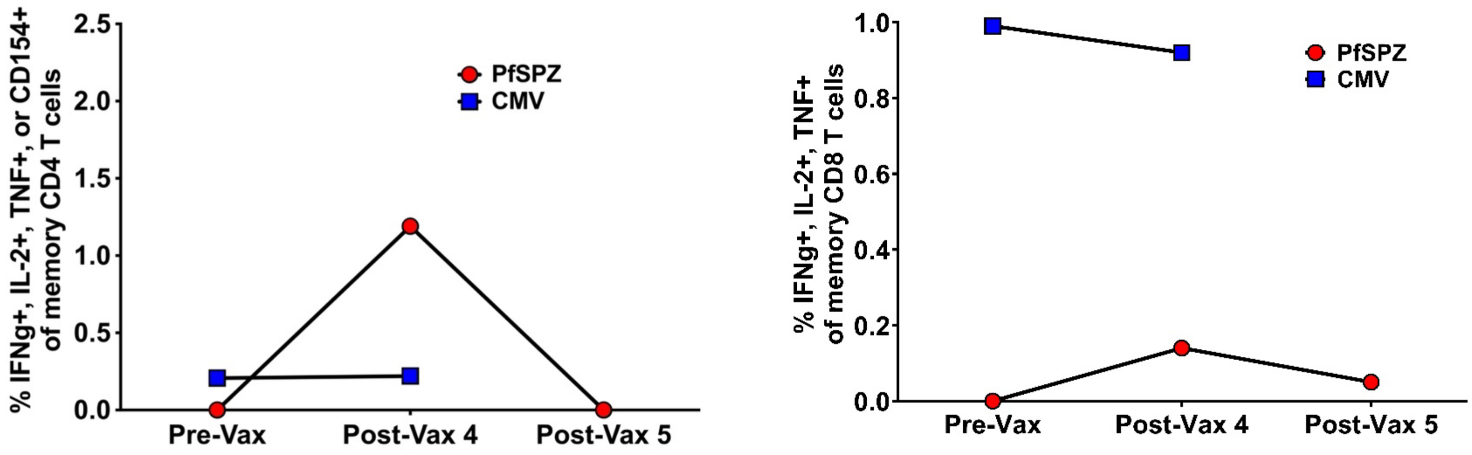

Participant U (HIV+, PfSPZ Vaccine)

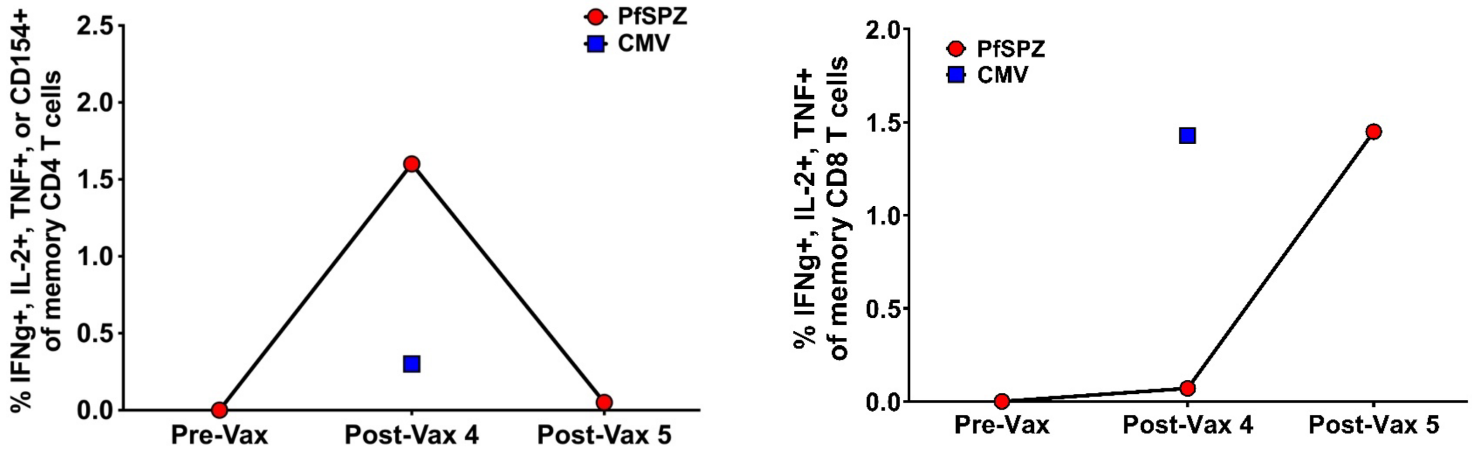

Participant M (HIV+, normal saline)

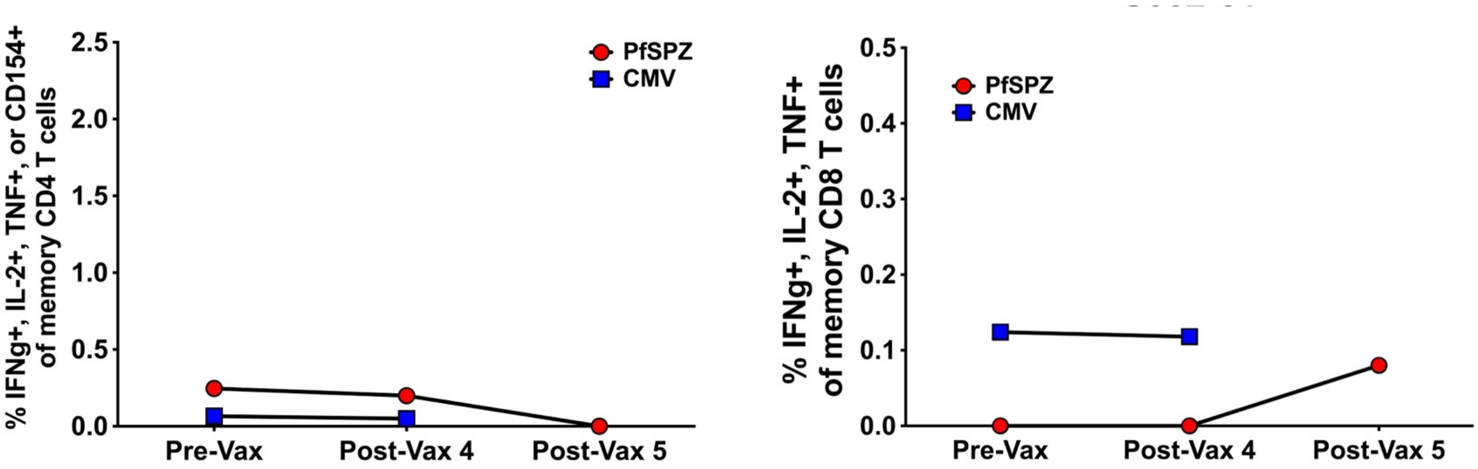

Participant P (HIV+, normal saline)

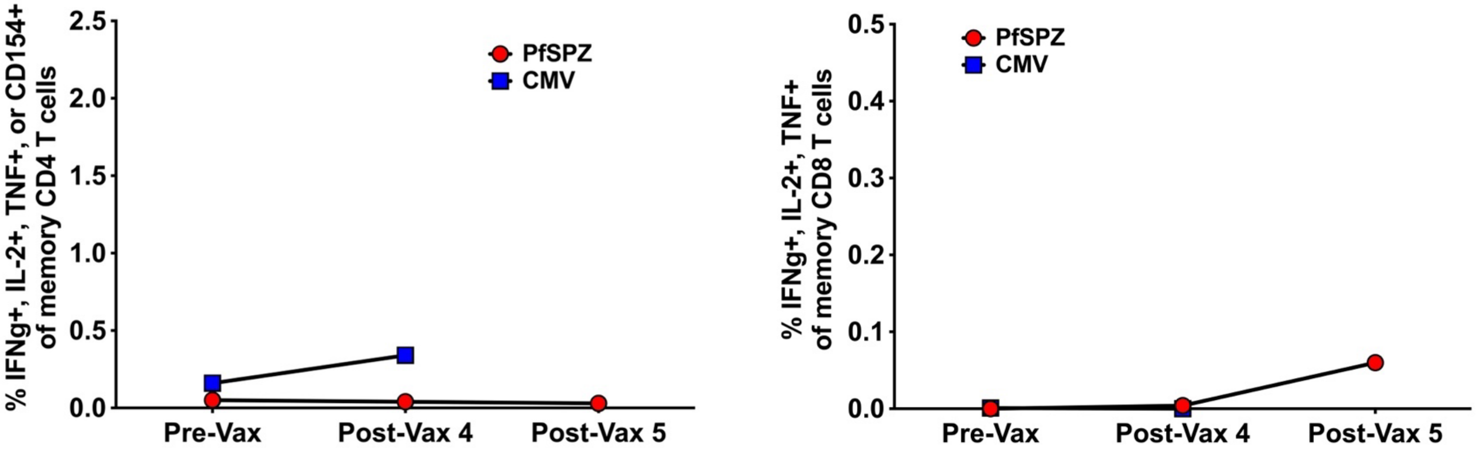

Participant T (HIV+, normal saline)

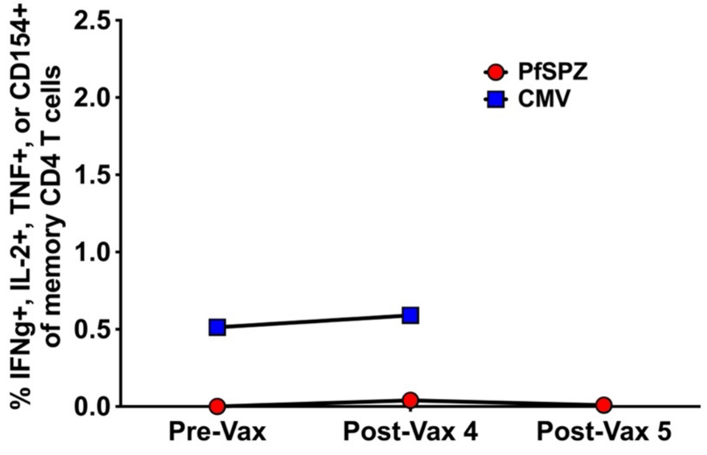

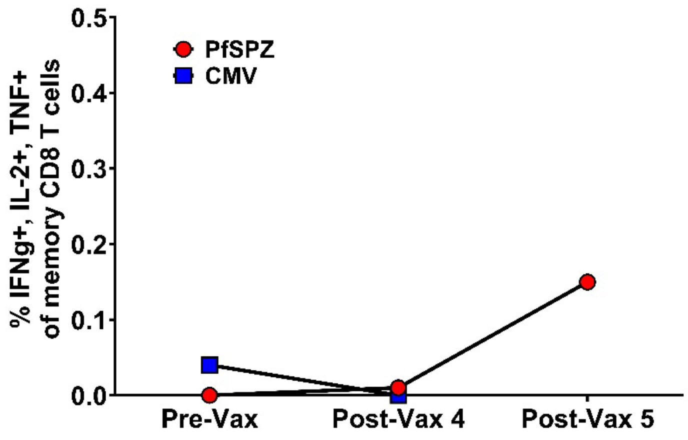

Figure S7. Flow cytometry staining panels and flow results for Vδ2+ and Vδ2- γδ T cells using PBMCs.

Staining panel

Antibody	Clone	Fluorochrome	Company	Catalog #
Ms anti Hu CD3	SP34-2	BB700 (AF700)	BD	566517
anti Hu CD4	SK3	eF450 (Pac Blue)	eBioscience	48-0047-42
Ms anti Hu CD8	SK1	APC-H7	BD	560179
Ms anti Hu VD2	B6	FITC	BD	555738
anti Hu gdTCR	5A6.E9	PE	Lifetechnologies	MHGD04
Ms anti Hu CD27	M-T271	PE Cy7	BD	560609
Ms anti Hu CD45RO	UCHL1	PE-CF594	BD	562299
Live Dead	na	Aqua (V500)	Invitrogen	L34957A

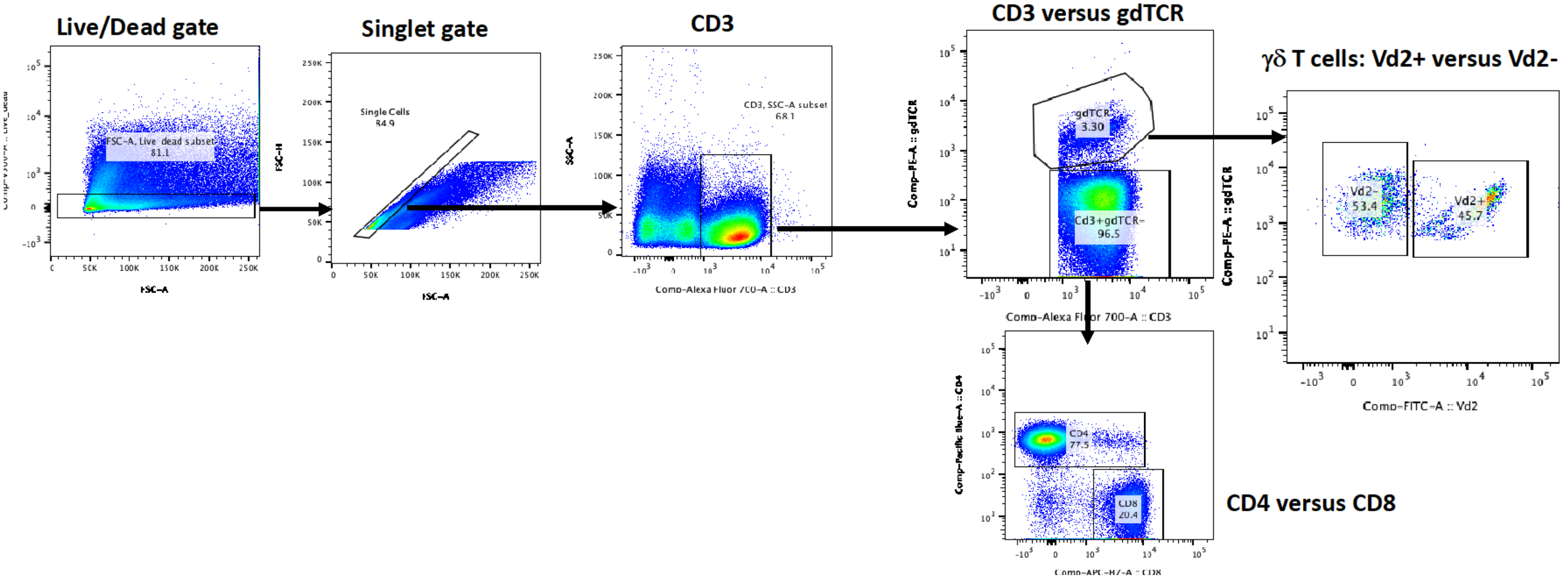

Supplement: Supplemental data [file jci-134-169060-s055.pdf]
